# Site fertility drives temporal turnover of vegetation at high latitudes

**DOI:** 10.1002/ece3.5778

**Published:** 2019-10-29

**Authors:** Tuija Maliniemi, Konsta Happonen, Risto Virtanen

**Affiliations:** ^1^ Geography Research Unit University of Oulu Oulu Finland; ^2^ Ecology and Genetics Research Unit University of Oulu Oulu Finland; ^3^ Department of Geosciences and Geography University of Helsinki Helsinki Finland; ^4^ Department of Physiological Diversity Helmholtz Center for Environmental Research – UFZ Leipzig Germany; ^5^ German Centre for Integrative Biodiversity Research (iDiv) Halle‐Jena‐Leipzig Leipzig Germany

**Keywords:** community stability, dynamic macroecology, long‐term research, plant community, plant strategies, site fertility, vegetation resurvey

## Abstract

Experimental evidence shows that site fertility is a key modulator underlying plant community changes under climate change. Communities on fertile sites, with species having fast dynamics, have been found to react more strongly to climate change than communities on infertile sites with slow dynamics. However, it is still unclear whether this generally applies to high‐latitude plant communities in natural environments at broad spatial scales. We tested a hypothesis that vegetation of fertile sites experiences greater changes over several decades and thus would be more responsive under contemporary climate change compared to infertile sites that are expected to show more resistance. We resurveyed understorey communities (vascular plants, bryophytes, and lichens) of four infertile and four fertile forest sites along a latitudinal bioclimatic gradient. Sites had remained outside direct human disturbance. We analyzed the magnitude of temporal community turnover, changes in the abundances of plant morphological groups and strategy classes, and changes in species diversity. In agreement with our hypothesis, temporal turnover of communities was consistently greater on fertile sites compared to infertile sites. However, our results suggest that the larger turnover of fertile communities is not primarily related to the direct effects of climatic warming. Furthermore, community changes in both fertile and infertile sites showed remarkable variation in terms of shares of plant functional groups and strategy classes and measures of species diversity. This further emphasizes the essential role of baseline environmental conditions and nonclimatic drivers underlying vegetation changes. Our results show that site fertility is a key determinant of the overall rate of high‐latitude vegetation changes but the composition of plant communities in different ecological contexts is variously impacted by nonclimatic drivers over time.

## Graphical abstract

Our results show that temporal vegetation turnover is larger on fertile than on infertile forest sites. However, the results suggest that the observed turnover is not primarily related to the direct effects of warmer climate. Compositional changes resulting in the larger turnover vary in different ecological contexts and are influenced by various nonclimatic drivers.

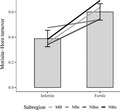

## 1 INTRODUCTION

A pioneering experimental study on British grasslands showed that community‐level changes under simulated climate change are dependent on site fertility and associated plant community composition. Communities on fertile sites changed rapidly in response to warming and water manipulations, while minor change was observed in communities on infertile sites (Grime et al., 2000). The explanation provided by the authors was relatively simple: Species exhibiting fast dynamics characterize fertile sites, whereas species with slow dynamics dominate in infertile sites and resisted short‐term experimental treatments. Thus, site fertility and associated plant functional types could determine the rate of plant community changes under climatic change. In the longer term, the communities on infertile sites showed more pronounced changes in composition but it was suggested that these communities would still respond relatively weakly to climatic changes (Fridley, Lynn, Grime, & Askew, 2016; Grime et al., 2008). These findings from British grasslands led to a development of a general view upon the role of site fertility as a key property influencing climate sensitivity of plant communities and might represent one of the few emerging generalizations on how plant communities respond to climate change (Harrison, Damschen, Fernandez‐Going, Eskelinen, & Copeland, 2015). However, it remains unknown whether these findings can be extrapolated to a wider range of natural ecosystems along broad geographical gradients. Such knowledge would be imperative for identifying links to experimental manipulations, ecosystem properties, and long‐term effects of ongoing climatic warming (Parmesan & Hanley, 2015).

The idea behind differential responsiveness of communities on fertile versus infertile sites emerge from plant life histories under different resource levels and evolved plant traits. These underlying foundations of communities have been strongly investigated from 1970s and pioneered by Grime (1974, 2001). The broad generalizations of evolved plant strategies are widely applicable in global temperate–boreal ecosystems (Grime, 2001; Grime, Hodgson, & Hunt, 2007). Yet, details in underlying processes and their mechanistic interpretations have been the subject of debate (Craine, 2005). However, it is not known whether plant strategy‐based approaches provide any useful predictions for plant community changes under climate warming across broader spatial scales. Despite advances in theory, modeling, and experimentation (Buri et al., 2017; Díaz et al., 2016; Pellissier et al., 2018; Reich, 2014), this remains an unresolved issue, recently been stressed also by Parmesan and Hanley (2015).

It is widely acknowledged that predicting the effects of climate change on plant communities is notoriously difficult due to complex relations between environmental conditions and multispecies assemblages (Fridley et al., 2016). Despite these difficulties, resource‐based plant strategy theories may allow some general predictions of climate change effects. In areas with short growing season, climatic warming can be expected to favor species that can take advantage of the prolonged growing season, which can be accompanied by improved availability of soil resources. Such conditions likely favor species with an ability to gain higher abundance under warmer conditions (Tilman, 1988), for instance, taller species that are superior competitors for light. This could further cause decreases of low‐statured species. In the context of Grime's (1974, 2001) plant strategy theory, this could mean an increase of species with the competitive strategy and a decrease of stress‐tolerant species. However, the responses of communities along fertility gradient are likely variable because of differing initial community compositions that result from distinct resource acquisition and conservation traits. Communities on fertile sites consist to a large extent of plants with fast dynamics (i.e., rapid lifecycles), while communities on infertile sites contain mainly species with slow dynamics (Grime, 1974, 2001; Reich, 2014). Long‐term observational studies at high latitudes have provided tentative evidence that the magnitude of plant community changes under recent climate warming is linked to a gradient from more productive (fertile) to less productive communities (Virtanen et al., 2010), while some broader scale comparative studies failed in finding any clear link between long‐term community changes and productivity (Kapfer et al., 2013). It is thus unsettled how fertility modulates the responses of local communities to climate change in different contexts.

In this study, we used forest vegetation resurvey data from several sites distributed over a latitudinal gradient to test whether site fertility is a general ecosystem property that influences the magnitude of plant community responses under long‐term climate warming. Specifically, we tested the hypothesis that long‐term compositional changes are consistently greater in communities on fertile than on infertile sites. We analyzed temporal turnover of communities and changes in plant strategy types, morphological groups, individual species, and species diversity, and examined how these changes are linked to trends in climatic and nonclimatic drivers.

## 2 MATERIALS AND METHODS

### 2.1 Study area and vegetation resurvey

The study area covers middle and northern boreal Finland (Ahti, Hämet‐Ahti, & Jalas, 1968; Appendix S1a). Infertile coniferous heath forests dominate for most of the latitudinal gradient and are replaced by mountain birch forests in the northernmost parts of the study area. Among the dominant infertile heath forests, fertile herb‐rich forests occur scattered throughout the gradient. The communities of fertile sites represent herb‐dominated vegetation types of forest understories, while dwarf shrubs, often with considerable cover of bryophytes and/or lichens, dominate the communities of infertile sites (Hämet‐Ahti, 1963; Sepponen, Laine, Linnilä, Lähde, & Roiko‐Jokela, 1982; Appendix S1b). The infertile and fertile forest sites differ not only in their field and ground layer species composition but also in terms of soil nutrients (Lahti & Väisänen, 1987; Sepponen et al., 1982).

We searched for historical data on the composition and structure of forest vegetation from 3–5 decades ago at fertile and infertile sites across the boreal zone in Finland. We found several forest vegetation studies from northern Finland that were sampled during 1957–1981 (Table 1) and encompass a south–north gradient from middle boreal to northern boreal zones (see Appendix S1a). More specifically, the surveyed sites represent the following four subregions (with abbreviations): the middle boreal zone (MB), the southern part of the northern boreal zone (NBs), the middle part of the northern boreal zone (NBm), and the northern part of the northern boreal zone (NBn; northern arboreal as defined by Ahti et al. (1968) and Hämet‐Ahti (1963)). All the original surveys contained detailed location information (e.g., specific area, description of the surroundings, elevation, aspect, specific vegetation type) allowing sufficiently accurate relocation of vegetation plots (Kopecký & Macek, 2015), which are thus regarded quasi‐permanent (sensu Kapfer et al., 2017). Relocation error and consequent effects of spatial heterogeneity on observed vegetation changes (see Verheyen et al., 2017) were further minimized as the original surveyor helped in relocating fertile sites in subregions MB, NBs, and NBm. For the infertile sites of same subregions, precisely drawn maps on plot locations existed. In subregion NBn, large plot size increased the possibility to relocate close to original plot location (Table 1). In 2013–2014, we relocated and resurveyed infertile and fertile forest sites, considering the original phenological date. In each original survey, the complete species composition (vascular plants, bryophytes, and lichens) was recorded using a sample plot method and species covers were estimated using percentage cover scales, which were applied in a similar manner during resurveys. The magnitude of human disturbance was estimated visually on site at plot scale and from land use raster maps classified by human activity for the surroundings using a radius of 100 m (further details in Walz & Stein, 2014) in ArcMap software (v. 10.2; ESRI).

**Table 1:** Information on resurveyed forest vegetation datasets

Sub region	Latitude/Longitude	Forest type	Soil type	Plot	Plot size (m)		Mean pH (range)	Years from original sampling	Data (published)
				*n*	shrub lr.	field lr.			
MB	64°N, 28°E	Herb‐rich	Mold	8	5 × 5	5 × 5	4.9 (4.0–5.4)	43–45 (1968–1970)	Kaakinen (1971)
	64°N, 28°E	Heath	Podzol	9	10 × 10	1 × 1^b^	3.9 (3.4–4.4)	33–34 (1980–1981)	Leinonen (1984)
NBs	66°N, 30°E	Herb‐rich	Mold	10	5 × 5	5 × 5	5.5 (4.0–6.0)	42–43 (1971–1972)	Kaakinen (1974)
	66°N, 29°E	Heath	Podzol^a^	10	30 × 30	1 × 1^c^	3.7 (3.5–3.8)	34–35 (1979–1980)	Mikkonen‐Keränen (1982)
NBm	68°N, 24°E	Herb‐rich	Mold	10	5 × 5	5 × 5	4.8 (4.3–5.2)	39 (1975)	Kaakinen (unpubl.)
	68°N, 27°E	Heath	Podzol	10	25 × 25	1 × 1^d^	3.7 (3.4–4.1)	34 (1980)	Lyytikäinen (1983)
NBn	69°N, 21°E	Herb‐rich	Mold	10	10 × 10	10 × 10	5.4 (4.8–6.0)	54–57 (1957–1960)	Hämet‐Ahti (1963)
	69°N, 21°E	Heath	Podzol	11	10 × 10	10 × 10	4.2 (3.8–4.6)	54–57 (1957–1960)	Hämet‐Ahti (1963)

Note

Subregion (MB = middle boreal, NBs = northern boreal/southern part, NBm = northern boreal/middle part, NBn = northern boreal/northern part), forest type (herb‐rich = fertile, heath = infertile), soil type (specified in original survey and resurvey), number of resurveyed vegetation plots, plot sizes used in cover estimations for shrub layer species (shrub lr.) and for field and ground layer species (field lr.), average soil pH (based on our own analyses of soil samples collected during resurvey), years from the original sampling and the original data.

a Partly paludified, peat‐like soil in the resurvey.

b Averaged from five 1 × 1 m vegetation grids.

c Averaged from two to four 1 × 1 m grids.

d Averaged from four 1 × 1 m grids.

Prior to statistical analyses on compositional changes, we explored the spatial distribution of study plots and disturbance levels. For this study, we included sites that met the following criteria: Each subregion had comparable number of vegetation plots per fertile and infertile sites (Table 1) and plots within each subregion covered about equal spatial extent. Thus, spatially strongly isolated plots (typically more than 100 km apart from the other plots) were left out. The individual plots within each site were situated at distances of 26–2,200 m (the average distance to nearest neighboring plot is 367 m). Moreover, we included only sites without signs of human disturbance (e.g., cuttings, forest management, constructions). Based on these preset selection criteria, a total of 78 plots were left for analyses (Table 1). Of these, 38 plots represent fertile herb‐rich forest communities and 40 plots infertile heath forest communities, which were distributed equally over the latitudinal MB–NBn gradient.

Before analyses, original and resurveyed species data were checked for consistency and some similar species were combined (*Dicranum fuscescens* with *flexicaule*, *Cladonia arbuscula* with *mitis*, and *Cladonia gracilis* with *ecmocyna*). Moss genus *Brachythecium* and liverwort genera *Lophozia* and *Barbilophozia* (excluding *Barbilophozia lycopodioides*) were treated at the generic level. The final dataset consisted of 281 species of which 151 vascular plant and 63 bryophyte species were found at fertile sites and 95 vascular plant, 45 bryophytes, and 34 lichen species at infertile sites. Species data were pooled into morphological plant groups (shrubs, dwarf shrubs, forbs, graminoids, pteridophytes, bryophytes, and lichens), and vascular plants were further categorized into Grime's (1974, 2001) plant strategy groups (competitors, competitive ruderals, ruderals, stress‐tolerant ruderals, stress‐tolerators, stress‐tolerant competitors, and generalists) after Pierce et al. (2017) whose globally calibrated CSR strategies allow comparisons between and within biomes.

### 2.2 Trends in climatic and nonclimatic drivers in the study area

Daily temperature and precipitation values over the study period were obtained from E‐OBS raster dataset (Haylock et al., 2008) for each site. Annual thermal sums were summed from daily temperatures of the growing season that exceeded the +5°C threshold. Growing season was considered to start and to end from the day following at least ten subsequent days with a daily average temperature ≥+5°C to mark the start and <+5°C to mark the end. Annual precipitation was summed from the daily data. Linear regressions were formed for annual thermal sums and precipitation at each sampling site using the original sampling year of each dataset as a starting point and resurvey year as an ending point. Changes in thermal sum (Δ°Cd) and annual precipitation (Δmm/year) were calculated by multiplying the slope of each regression by the number of years between the original survey and resurvey in each dataset. Thus, comparable change values in thermal sums and precipitation were gained for each dataset that vary in sampling years.

Reindeer (*Rangifer tarandus*) is the main herbivore in the study area excluding study sites in the subregion MB that is located outside reindeer herding districts. Data on annual reindeer numbers were obtained from Natural Resources Institute Finland. In the subregion MB, moose densities have been relatively high, with small populations of forest reindeer and roe deer (Siira, Keränen, & Heikkinen, 2009). The fertile sites in subregion MB have likely been grazed by free‐ranging cattle long ago, but this activity ended before the first vegetation survey (Happonen, 2016; Kaakinen, 1974). At a European scale, northernmost Finland receives the lowest N deposition (Harmens et al., 2011), but it cannot be ruled out that the changes in atmospheric chemistry and associated nutrient addition or changes in sulfur deposition would have some impacts on vegetation in the longer term (Fowler et al., 2007). As our sampling sites represent forested sites with minimal direct human disturbances, changes in understorey composition and structure can be attributed to successional development toward more closed canopy. Alternatively, canopies may have opened up due to reindeer browsing preventing tree regeneration or insect outbreaks causing tree mortality. To document possible changes in overstoreys between the surveys, data from overstorey structure and cover were obtained from the original studies, and repeated samplings for the same parameters were conducted.

### 2.3 Statistical analyses

The magnitude of compositional turnover in plant communities over time was quantified by calculating Morisita–Horn similarities between each pair of original and resurveyed plot using the package *vegetarian* (Charney & Record, 2015) in R (R Development Core Team, 2018). The Morisita–Horn similarity index was chosen because it is not strongly sensitive to variation in species richness (Chao, Chazdon, Colwell, & Shen, 2006), that is affected to some degree by the varying plot sizes between the study sites (see preliminary analyses in Appendix S2), and to minimize the errors due to observer bias (Kapfer et al., 2017). A Bayesian linear regression model with normally distributed errors was fitted to test the effects of site fertility on the magnitude of compositional turnover using package *brms* (Bürkner, 2019), an interface to the Bayesian modeling framework Stan (Carpenter et al., 2017). This package was also used to fit the following models. In additional models, linear, nonlinear, and interactive effects of sampling time (e.g., the temporal difference between the original survey and resurvey) were further included as covariates to test if they affect the magnitude of turnover. Nonlinear effects were included as thin‐plate splines and their interaction with fertility as a factor smooth interaction. Plot size was not independent of sampling time and thus was left out from the models. Different models were ranked using pareto‐smoothed importance sampling leave‐one‐out cross‐validation (PSIS‐LOO, Vehtari, Gelman, & Gabry, 2017). The results of the best model were visualized by plotting the posterior distribution of average turnover in fertile and infertile forests. To further visualize temporal trends in compositional change in each study site, nonmetric multidimensional scaling (NMDS) ordination with Bray–Curtis distances and plot‐scale species data were applied from package *vegan* (Oksanen et al., 2018).

The cover of morphological plant groups and specified CSR strategy groups varied considerably between fertile and infertile sites and between subregions (see Appendix S3). Thus, temporal changes in the absolute cover of morphological plant groups and specified CSR strategy groups are presented for each site as means, with bootstrapped confidence intervals. For displaying and analyzing changes in relative coverages of main strategy classes (C, S, and R), their proportional values (%) were first assigned for all species according to Pierce et al. (2017). Subsequently, main strategy values of each species were weighted by their cover (%) in each plot. Weighted values of C, S, and R strategies were summed up for each class, which were then divided by the total cover of the plot to achieve the relative proportions of strategy class covers. The covers of discrete CSR‐classes were thus effectively transformed into three continuous variables. A multivariate Bayesian regression model with normally distributed errors was built to test if the proportional values of main strategy classes had changed over time in fertile and infertile sites. Time (old vs. new), fertility type (fertile vs. infertile), and their interaction were included as fixed effects. Plot identity was specified as a random intercept to account for repeated measurements. The plot‐specific random effects and residuals were allowed to covary between strategy classes, as a covariance matrix was fitted for both. The proportional covers of strategy classes were logit‐transformed before modeling to avoid bias due to boundaries in proportional data and to ensure that model residuals were approximately normal. The model was fit only for main strategy class C and S because of identifiability—the relative cover of R must always be 100% − (C + S). The results of the model were visualized by calculating average proportional cover of each main strategy class for each time period from the posterior distribution of the multivariate model. Error variance and plot‐specific random effects were omitted from the visualizations.

Changes in species diversity per plot over time were quantified by calculating effective number of species for Simpson diversity measures (Jost, 2006, 2007) using the package *vegetarian*. The Simpson diversity measure gives more weight to more abundant species and thus has the same benefits as the Morisita–Horn index, used for analyzing temporal turnover. The effective number of species is the number of equally abundant species needed to achieve the diversity measure and thus corresponds to a unit conversion of Simpson's diversity index from probability to species (Jost, 2006, 2007). Effective species numbers were calculated for vascular plants, bryophytes, lichens, and for the total number of species. Bayesian regression models with normal error distributions were built for log(x + 1)‐transformed total, vascular, and bryophyte species diversity. Time, fertility type, and their interaction were included as fixed effects and plot identity as a random effect to account for repeated measurements. Because lichens were found only at infertile sites, their diversity was modeled only with time as fixed and plot identity as a random effect. Model results were visualized as the posterior distributions of average Simpson diversity in fertile and infertile sites at the two time periods. Plot‐specific random effects and residual variance were not considered in the visualizations.

Lastly, the direction of beta diversity changes was analyzed, because temporal turnover within each site can be due to either rising compositional similarity (homogenization) or dissimilarity (heterogenization). First, a multiple‐sample Morisita–Horn index was calculated for each site in the original survey and resurvey, using the package *vegetarian*. Resulting value ranges from zero (no turnover between samples) to one (complete turnover between all samples). The sign of beta diversity change was then calculated as a difference between each pair of original and resurveyed sites (total *n* = 8). The multiple‐sample Morisita–Horn index was used in the calculations to make analyses comparable to those of temporal turnover and alpha diversity change.

## 3 RESULTS

Thermal sums showed relatively similar increasing long‐term trends throughout the study sites (Appendix S4a,b), rising between +182 and +253°Cd over the study period. An exception was the northernmost subregion NBn, where a minor increase (+71°Cd) was recorded. Annual precipitation increased between +30 and +153 mm but decreased slightly (−10 mm) at infertile site in subregion NBm (Appendix S1a). Reindeer grazing pressure increased over the study period but showed considerable fluctuations especially in the northernmost subregion NBn (Appendix S4c). Estimates of overstorey coverage changed over time in both fertile and infertile sites (Appendix S5). Substantial increases (MB and NBm) or decreases (NBs and NBn) in canopy cover occurred at fertile sites, whereas more modest decreases took place at infertile sites (canopy cover estimate was not available for the original survey in subregion NBm).

**Figure 1:**
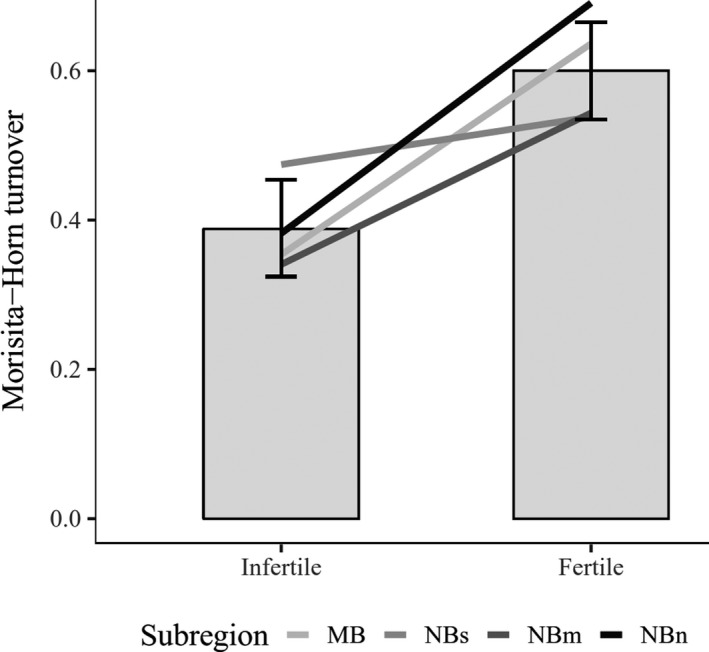
Simulated Morisita–Horn turnover from a Bayesian regression model for fertile and infertile sites with 95% credible intervals. The observed means of each subregion during the original survey and resurvey are indicated as gray‐scale lines. Turnover is Morisita–Horn dissimilarity index (1‐Morisita–Horn index) that was calculated for each pair of original and resurveyed vegetation plot

**Figure 2:**
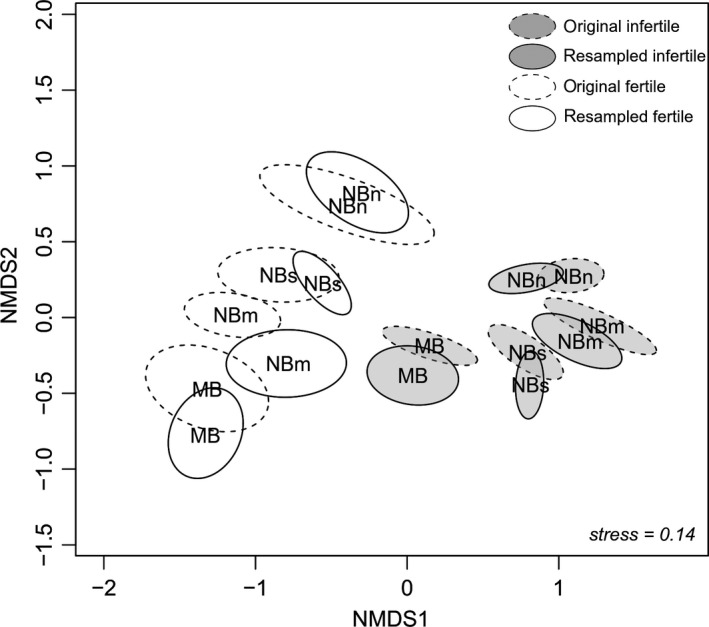
Nonmetric multidimensional scaling (NMDS) ordination grouped by original and resurveyed communities on fertile and infertile sites in subregions middle boreal zone (MB), northern boreal zone/southern part (NBs), northern boreal zone/middle part (NBm), and northern boreal zone/northern part (NBn). Labels within the confidence ellipses show the locations of group centroids

In agreement with our predictions, the magnitude of compositional turnover between original and resurveyed vegetation plots was greater on fertile than on infertile sites across all subregions (Figure 1). The final model included only site fertility (point estimate −0.21 with credible intervals between −0.30 and −0.12), and sampling time (e.g., the period between original survey and resurvey) had no effect on turnover (but see Appendix S6). The model explained c. 22% of the total variation (using Bayesian *R* ^2^, Gelman, Goodrich, Gabry, & Vehtari, 2019). The NMDS ordination showed that the compositional distinctness between communities on fertile and infertile sites largely remained over time (Figure 2). However, the composition of fertile communities shifted slightly toward infertile communities in subregions NBs and NBm, whereas the composition of infertile communities shifted a little toward fertile communities in subregion NBn.

**Figure 3:**
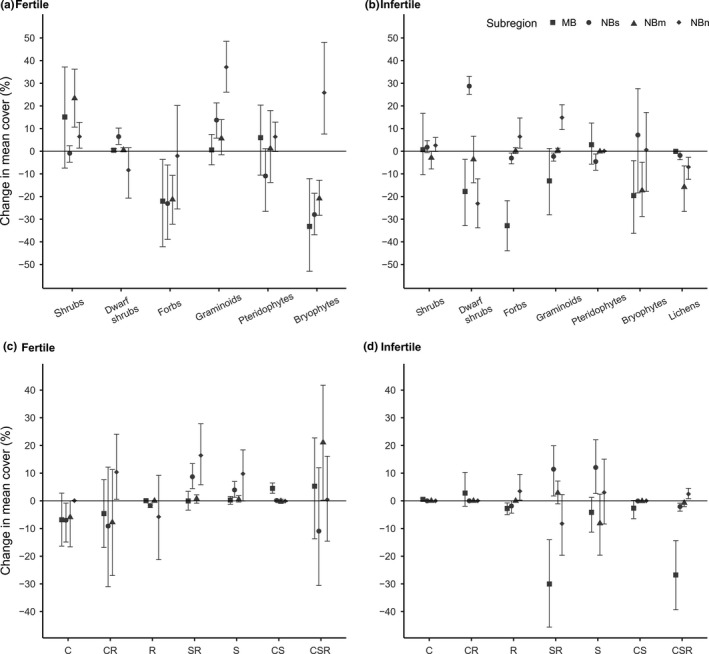
Observed mean change in the absolute % ‐cover of morphological plant groups in (a) fertile and (b) infertile sites and (c) plants assigned into plant strategy classes (C, competitors; CR, competitive ruderals; R, ruderals; SR, stress‐tolerant ruderals; S, stress‐tolerators; CS, competitive stress‐tolerators; CSR, generalists) in fertile and (d) infertile sites in each subregion. Error bars represent bootstrapped 95% confidence intervals. Plant group and strategy class covers (%) in original survey and resurvey are shown in Appendix S3

In general, changes in plant group covers on fertile or infertile sites showed no clear consistency along the latitudinal gradient (Figure 3a,b). However, there was a tendency toward increasing shrub and graminoid cover and decreasing forb cover in fertile sites. Bryophytes decreased strongly except in subregion NBn where a clear increase took place (Figure 3a; see Appendix S7 for species‐specific changes). Regionally variable changes in plant group covers were detected also in infertile sites. In subregion MB, especially dwarf shrubs, forbs and bryophytes decreased. Clear increase of dwarf shrubs took place in subregion NBs. Bryophytes and lichens decreased in subregion NBm. In subregion NBn, dwarf shrubs and lichens decreased but forbs and graminoids increased (Figure 3b; species‐specific changes in Appendix S8).

**Figure 4:**
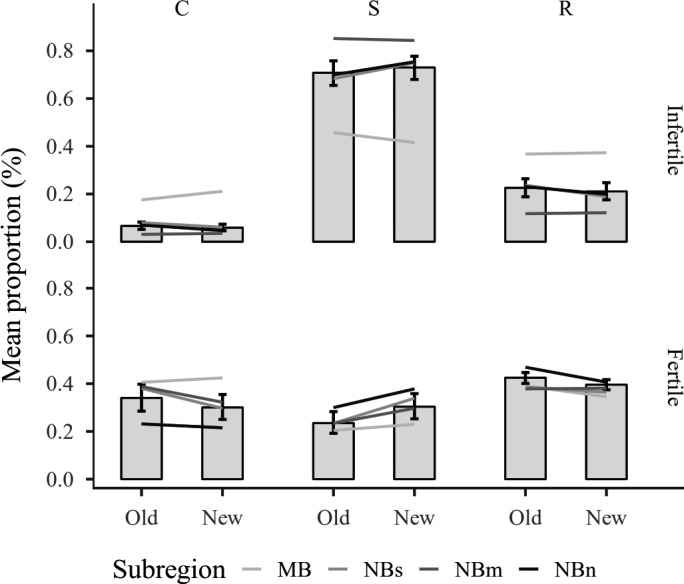
Posterior simulations of main strategy class (C, S, and R) proportions for fertile and infertile sites with 95% credible intervals. The observed means of each subregion during the original survey and resurvey are indicated as gray‐scale lines

Similarly, mostly inconsistent and only few clear changes were observed in covers of specified plant strategy groups among studied sites. In fertile sites, our results generally suggested a decrease of either C, CR, or R‐strategists and an increase of either SR, S, or CS‐strategist (Figure 3c), whereas in infertile sites patterns were less clear. SR‐ and CSR‐strategists decreased clearly in subregion MB while SR‐ and S‐strategists increased in subregion NBs (Figure 3d). Bayesian posterior simulations of proportional main strategy class (C, S, R) changes in fertile sites revealed that competitive strategy (C) clearly decreased and stress‐tolerators (S) increased over time (Figure 4). The covers of main strategy classes remained stable in infertile sites (see also conventional CSR‐triangles in Appendix S9). The fixed effects explained c. 60% of the variation in strategy class proportions, while random effects explained further c. 20%.

**Figure 5:**
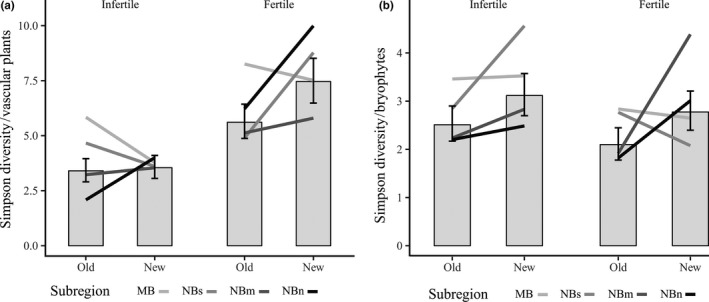
Simulated (a) vascular plant species diversity and (b) bryophyte species diversity for fertile and infertile sites with 95% credible intervals. Diversity measures are effective numbers of Simpson diversity. The observed means of each subregion during the original survey and resurvey are indicated as gray‐scale lines (specific values in Appendix S10c)

Changes in species diversity showed no constant trends between subregions and were rather variable over time. Vascular plant diversity of fertile sites generally increased but showed considerable variation among subregions (Figure 5a). Bryophyte diversity mainly increased on all sampled sites except for the fertile sites of the southern subregions (Figure 5b). Lichen diversity, measured only from infertile sites, remained constant (Appendix S10a). Because of the diverging trends between plant group diversities, only a weak increase was seen in total diversity (Appendix S10b). The fixed (fixed + random) effects explained ~20% (~35%) of total diversity, ~35% (~50%) of vascular plant diversity, ~10% (~20%) of bryophyte diversity, and ~2% (~5%) of lichen diversity. Regarding all Bayesian models, neither default model diagnostics nor visual inspection of the Markov chains and the posterior predictive distributions indicated problems in model convergence or serious model misspecification.

Within‐site beta diversity was higher in fertile sites during both surveys across the study area (Table 2). However, neither the composition of fertile nor infertile communities showed general trend along latitudinal gradient toward homogenization or heterogenization over time. Instead, the direction of compositional change varied locally.

**Table 2:** Beta diversity, as a community turnover rate with standard error inside brackets, within studied communities in the original survey (*β*_old_) and resurvey (*β*_new_) on fertile and infertile sites in each subregion

Subregion	Fertile sites			Infertile sites		
	*β*_old_	*β*_new_	Δ*β*	*β*_old_	*β*_new_	Δ*β*
MB	0.198 (0.012)	0.283 (0.021)	0.085	0.091 (0.006)	0.130 (0.013)	0.039
NBs	0.093 (0.007)	0.078 (0.005)	−0.015	0.073 (0.007)	0.064 (0.005)	−0.009
NBm	0.078 (0.006)	0.131 (0.008)	0.053	0.083 (0.006)	0.040 (0.006)	−0.043
NBn	0.147 (0.009)	0.156 (0.008)	0.009	0.029 (0.002)	0.054 (0.005)	0.025

Note

Rates range from zero (no turnover between sample plots of a site) to one (complete turnover between samples). Difference between old and new turnover value (Δ*β*) indicates whether community compositions show signs toward heterogenization or homogenization.

## 4 DISCUSSION

In this broad‐scale, long‐term, and multisite observational study, we tested whether the level of site fertility consistently influenced the compositional changes of forest understorey plant communities under contemporary climate change. Based on comparisons of communities sampled recently and decades ago, our results support the prediction that compositional changes in plant communities are greater on fertile than on infertile sites. This was indicated by the larger compositional turnover of fertile communities over time. This result is parallel with the experimental and observational evidence from other ecosystems (Grime et al., 2000; Harrison et al., 2015). However, our results suggest that the larger turnover of fertile communities is not generally related to direct effects of climate warming. Moreover, in terms of individual species, major plant groups, plant strategy groups, and species diversity measures, relatively inconsistent vegetation changes took place among subregions and between communities on fertile and infertile sites despite a rather uniform climate warming trends across the study area. This further emphasizes the essential role of baseline environmental conditions and nonclimatic drivers underlying vegetation changes (Bernhardt‐Römermann et al., 2015). Furthermore, we underpin the importance of long‐term resurvey studies from multiple sites for a development of a better understanding of the complexities between long‐term dynamics of plant communities under climatic and nonclimatic environmental changes (Verheyen et al., 2017).

The greater compositional turnover on fertile sites over time likely results from the contrasted plant functional types between the communities on fertile and infertile sites. On fertile sites, relatively fast‐growing herbaceous plants dominate, and plants with competitive strategies (C, CR) or “fast” dynamics (Reich, 2014) are relatively abundant. On infertile sites, in contrast, communities are composed virtually entirely of more stress‐tolerant species (S‐ and SR‐strategists) or plants with “slow” dynamics. Despite the long‐term timescale, the difference in the magnitude of community changes between fertile and infertile sites is not as striking as shown by the 5‐year experimental treatments by Grime et al. (2000), which is likely due to rather late successional stages of studied communities. Moreover, Grime et al. (2008) found that in the longer term, plant communities also on infertile sites showed more turnover. Therefore, it is possible that site fertility better predicts the rate of long‐term community changes rather than specific long‐term compositional outcomes.

Given that all subregions experienced a warming trend and often increase in precipitation, we expected that species with the competitive strategy (e.g., tall herbs, shrubs) increased at the expense of competitively inferior species (e.g., low‐growing species) through compensatory competition (Tilman, 1999). Contrary to this expectation, we did not observe a consistent climate‐driven increase of competitive plants across subregions, albeit slight increases of individual competitive species (e.g., woody species) were detected locally. In addition, the expected decrease of least competitive stress‐tolerators was not consistent across subregions, although low‐growing cryptogams (bryophytes and lichens) decreased on many sites. The decreasing tendency in the relative proportion of main competitive strategy class in fertile sites and the simultaneous increase in the proportion of stress‐tolerant strategy do not match with findings of De Frenne et al. (2015), who found experimental evidence that warmer conditions with increased light could favor tall (competitive) plants. However, we argue that the observed long‐term trajectories of natural plant communities underclimatic warming trends may be more variable than in short‐term experiments. The absence of direct climate‐driven vegetation changes corresponds to analogous study from northern boreal forests of Sweden, where Hedwall and Brunet (2016) found no temperature‐related vegetation changes. A growing amount of evidence is indicating that compositional shifts in forests may lag behind climatic warming due to the microclimatic buffering effect from the canopy layer (De Frenne et al., 2013, 2019) or unchanged snow cover conditions (Kreyling, Haei, & Laudon, 2012). Thus, stronger compositional shifts and more direct responses to warming may emerge as climate change proceeds.

The lack of general trends in changes of plant morphological and specified strategy groups, individual species and diversity measures strongly suggests that compositional changes in forest understorey communities are linked to nonclimatic drivers. We emphasize that changes in overstorey structure, insect outbreaks, or reindeer grazing likely underlie the observed local changes in communities and interplay with recent climatic changes. Essentially, as has been observed elsewhere (Bernhardt‐Römermann et al., 2015; Hédl, Kopecký, & Komárek, 2010; Prach & Kopecký, 2018), these nonclimatic drivers may explain some of the most evident “anomalies” in vegetation changes. In addition, compositional shifts within vegetation types can be highly variable in different geographic contexts in general (see, for example, Maliniemi, Kapfer, Saccone, Skog, & Virtanen, 2018). Canopy shading increased particularly in fertile site of subregion MB, and this may have contributed to the increasing abundance of some shade‐tolerant species (a large fern *Dryopteris expansa*) and decline of light‐loving mosses (e.g., *Hylocomiastrum pyrenaicum*). In contrast, in the northernmost subregion NBn, the overstorey (entirely formed by mountain birch) encountered defoliating outbreaks of autumnal moth (*Epirrita autumnata*) in 2004–2006, c. 10 years before the resurvey (and less also later). These events may have reduced canopy shading and, along with relatively high reindeer grazing, may have led to the increasing abundance of a range of light‐loving and/or grazing tolerant graminoids (e.g., *Deschampsia*, *Poa*) and bryophytes (also Kopisto, Virtanen, Pekkanen, Mikkola, & Kauhanen, 2008). Thus, this reduction in canopy shading could also have accelerated changes in understorey communities (see also De Frenne et al., 2015; Valladares, Laanisto, Niinemets, & Zavala, 2016). Most likely, also reindeer grazing influences the structure of main plant strategies over time. Grazing buffers against climate‐driven increase of tall shrubs and certain forbs (Olofsson et al., 2009; Pajunen, Virtanen, & Roininen, 2008; Saccone, Pyykkonen, Eskelinen, & Virtanen, 2014), which have generally high shares of competitive strategy, and maintains disturbance that is reflected in the relatively large proportion of R‐strategy within studied communities. Thus, the larger turnover on fertile sites may be related to secondary succession due to continuous disturbance (Pierce et al., 2017). An exception to this is the subregion MB that locates outside the reindeer grazing districts. Here, slight increase of competitive species and the release from long‐term grazing by cattle may have enhanced the turnover (e.g., Czortek et al., 2018). It is possible that the impact of nonclimatic drivers discussed above is more pronounced on fertile than on infertile sites, and this could have contributed to the observed differences in the overall magnitude of community changes.

Even though infertile communities showed in general less temporal turnover, rather substantial subregion‐specific changes in their species and species‐group abundances had occurred. Among infertile communities, the largest turnover had taken place in the subregion NBs. Here, the decreasing abundance of species typical for drier heaths (e.g., *Vaccinium vitis‐idaea* and *Deschampsia flexuosa*), the strong increase of dwarf shrub abundance (e.g., *Empetrum hermaphroditum*, *Vaccinium uliginosum*), and the immigration and expansion of *Sphagnum* and *Polytrichum* mosses indicate the development of a paludified community. This is further supported by the discovery of partly peaty soil during the resurvey. These patterns match well with a relatively strong increase in precipitation and possibly increased levels of humidity and soil moisture. Thus, even if communities on infertile sites, with slow‐growing stress‐tolerant plants, resist better against short‐term climatic changes, they may still strongly react to long‐term changes in climate regimes. Despite their characterization as stress‐tolerators with slow growth (Grime, Rincon, & Wickerson, 1990), mosses with clonal growth and high spore‐dispersal capacity might be especially responsive (see also Becker Scarpitta, Bardat, Lalanne, & Vellend, 2017). In the time scales of two or more decades, even slow‐growing species can attain a dominant position (Saccone et al., 2014).

Our resurvey analyses revealed that among all studied sites local species diversity had either increased or remained stable over the past few decades. However, we did not observe any pattern in the directional beta diversity changes (homogenization‐heterogenization) neither on fertile nor on infertile sites. The decrease of compositional variation in fertile communities in subregion NBs, which is also associated with a change from formerly herb‐rich (*Geranium*, *Filipendula*) to recent graminoid‐rich (*Elymus*, *Melica*) composition, may be indicative of grazing‐driven increases of species diversity and compositional homogenization. In this subregion, grazing increased between 1980 and 2010, and studies elsewhere have shown that increased grazing pressure can contribute to preservation of high local‐scale species diversity (Chollet, Baltzinger, Le Saout, & Martin, 2014; Kaarlejärvi, Eskelinen, & Olofsson, 2017). Even so, relatively intense grazing pressure can also lead to more homogeneous community composition (Rooney, 2009). A prominent increase in species diversity was also observed in fertile sites of subregion NBn, likely resulting from grazing and increased light availability as mentioned above, but contrary to fertile sites in subregion NBs, no signs of homogenization were seen. This can be due to high initial species richness and relatively large species pool of the area, buffering the community against homogenization. The increased species richness in reindeer‐grazed subareas NBs, NBm, and NBn is related to increasing abundance of local graminoid and bryophyte species.

Our resurvey approach analyzing long‐term compositional changes across a broad‐scale gradient revealed both generalities and local context‐dependent responses of plant communities subjected to recent climate changes and nonclimatic drivers. Plant strategy types may have a primary value in generating firsthand predictions on expected shifts in plant species and functional structure. However, the deviations from general hypotheses are crucial for finding out the potential complexities related to prediction of vegetation changes under climate change. Despite the various compositional changes in studied northern plant communities, our study suggests that site fertility can be regarded as a general ecosystem property that predicts the magnitude of compositional turnover in high‐latitude plant communities over time.

## CONFLICT OF INTEREST

None declared.

## AUTHOR CONTRIBUTIONS

TM and RV initiated the study. KH and TM analyzed the data. TM led the writing of the manuscript. All authors contributed to data collection, designing the analyses and reading and revising the manuscript.

## Data Availability

Data used in this study will be deposited in BioTIME (Global database of biodiversity time series, biotime.st‐andrews.ac.uk) and is available upon request from the corresponding author (tuija.maliniemi@oulu.fi).
