# Supplementary material for: Site fertility drives temporal turnover of vegetation at high latitudes
Source: Ecol Evol. 2019 Oct 29;9(23):13255–66. doi: 10.1002/ece3.5778 (PMC6912880; doi:10.1002/ece3.5778)
Supplement: Supplementary file 1 [file ECE3-9-13255-s001.docx]

**Supplementary material**

**Appendix S1. a)** A map of the study area showing site locations, bioclimatic zonation (drawn after Moen 1999 and modified after Virtanen et al. 2016) and changes in thermal sum (Δ ^o^Cd) and annual precipitation (Δ mm) between each site-specific sampling periods. Names of subregions refer to middle boreal (MB), southern part of northern boreal (NBs), middle part of northern boreal (NBm) and northern part of northern boreal zone (NBn) with site type ‘fer’ for fertile and ‘inf’ for infertile sites. **b)** Photos represent both site types in each subregion (Photos: Tuija Maliniemi, Risto Virtanen).

**a) b)**

**
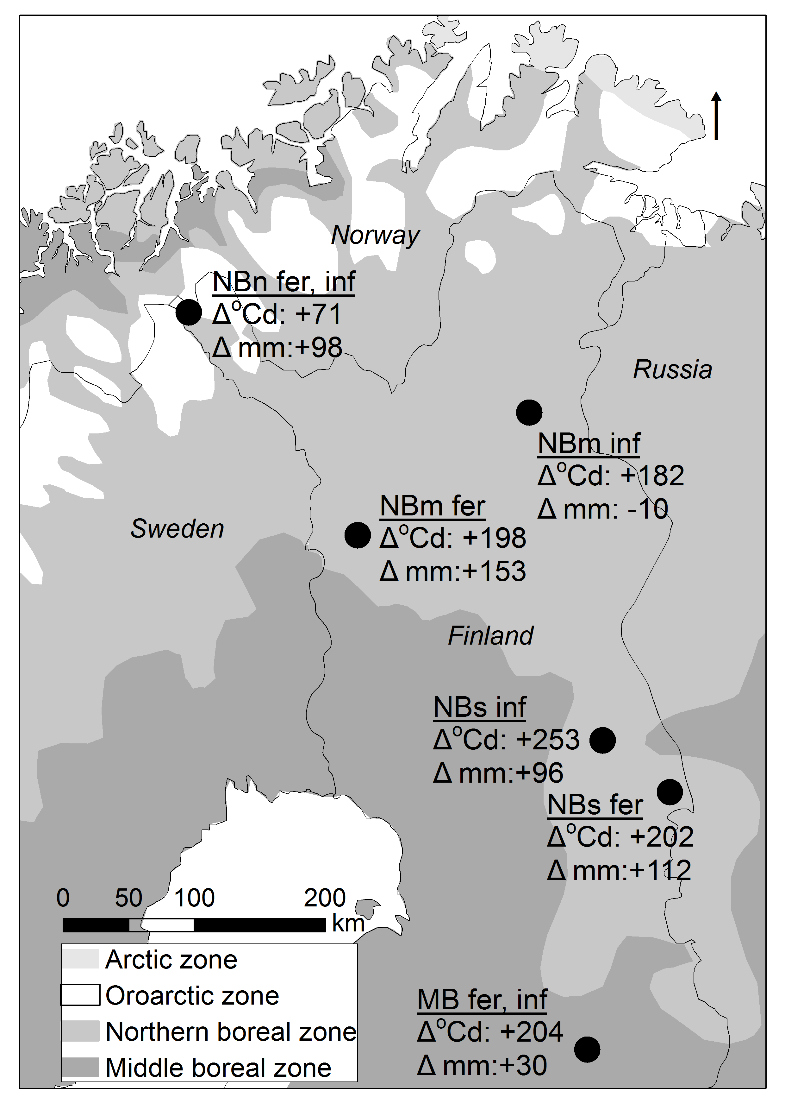

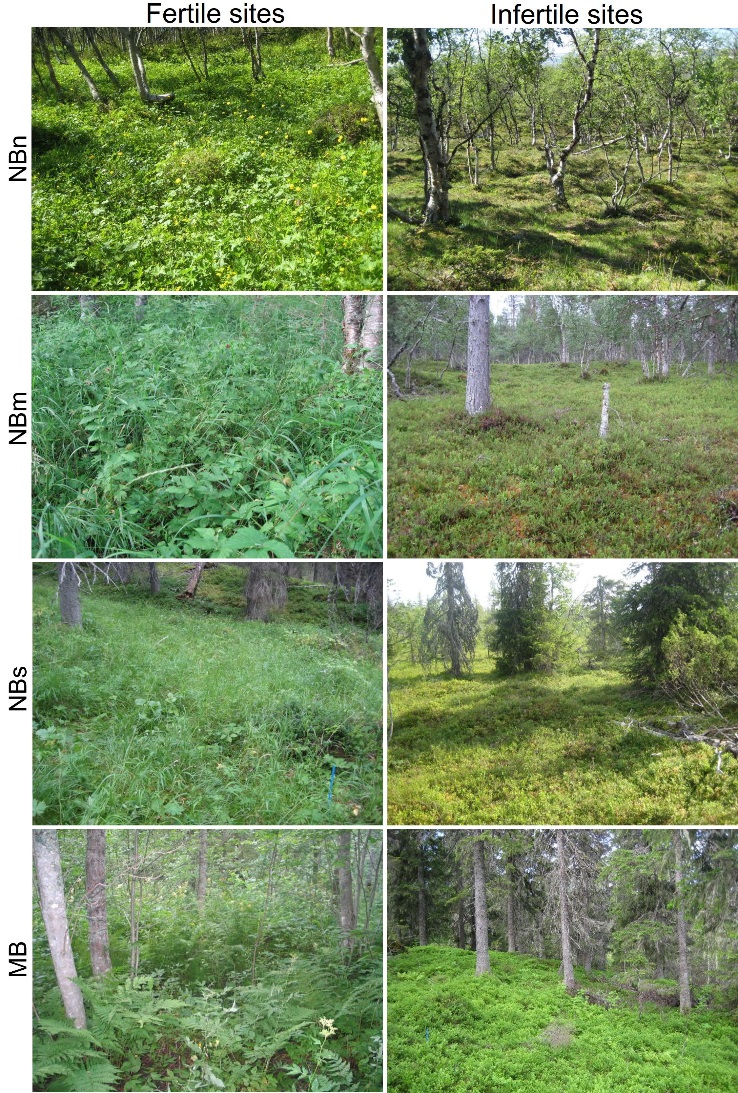
**

References:

Moen, A. (1999). *National atlas of Norway: vegetation*. Norwegian Mapping Authority: Hønefoss.

Virtanen, R. et al. (2016). Where do the treeless tundra areas of northern highlands fit in the global biome system: Towards an ecologically natural subdivision of the tundra biome. *Ecology & Evolution, 6,* 143–157.

**Appendix S2.** The effect of plot size to species diversity at each sampling period was tested for the whole data set and separately for both fertile and infertile sites using generalized linear models. The effect was tested separately for **a)** field and **b)** shrub layer species as different plot sizes were used to estimate their abundances (see Table 1 in the main document). Note that species diversity is prominently lower in the shrub layer than field layer. The only positive significant effect of plot size to species diversity was found in field layer species diversity of resurveyed fertile communities.


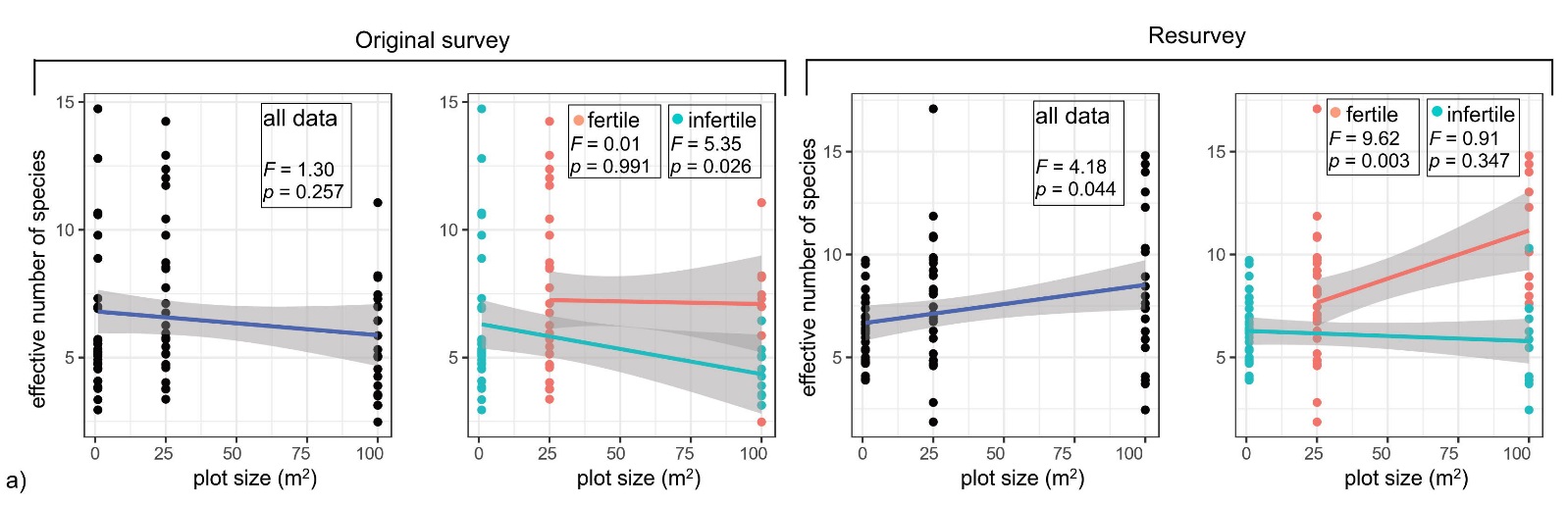


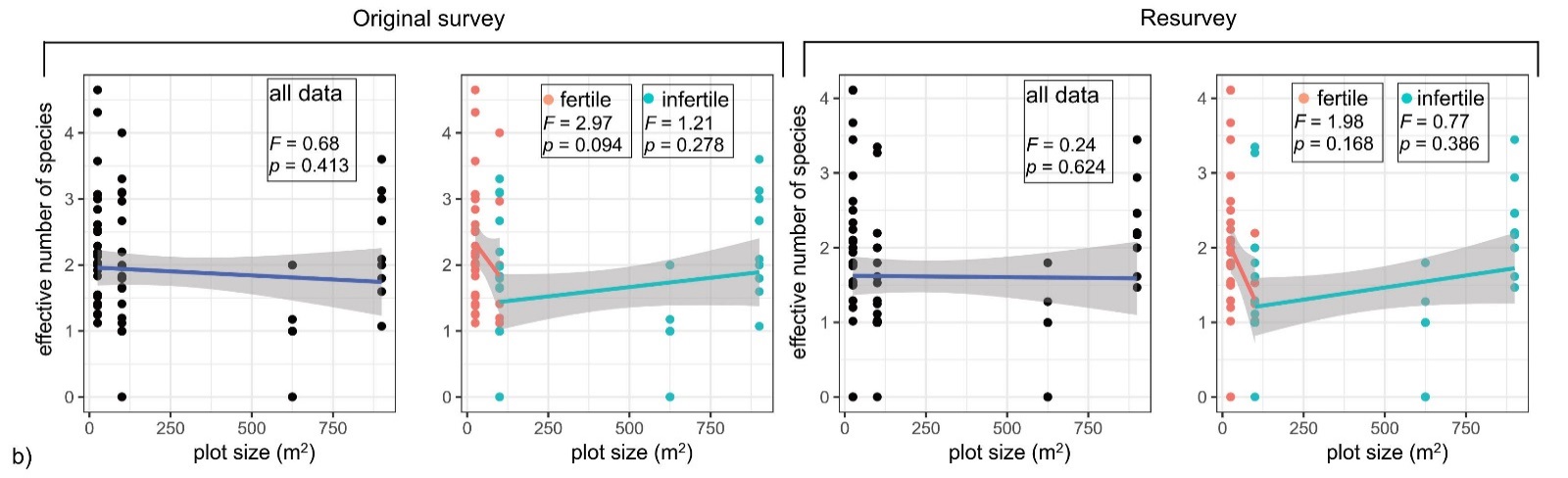


**Appendix S3.** Mean covers of **a)** morphological plant groups in fertile and **b)** infertile sites and **c)** plant strategy theory (CSR) classes in fertile and **d)** infertile sites in each subregion during original survey and resurvey. Note that the y-axis scales are different between fertile and infertile sites.


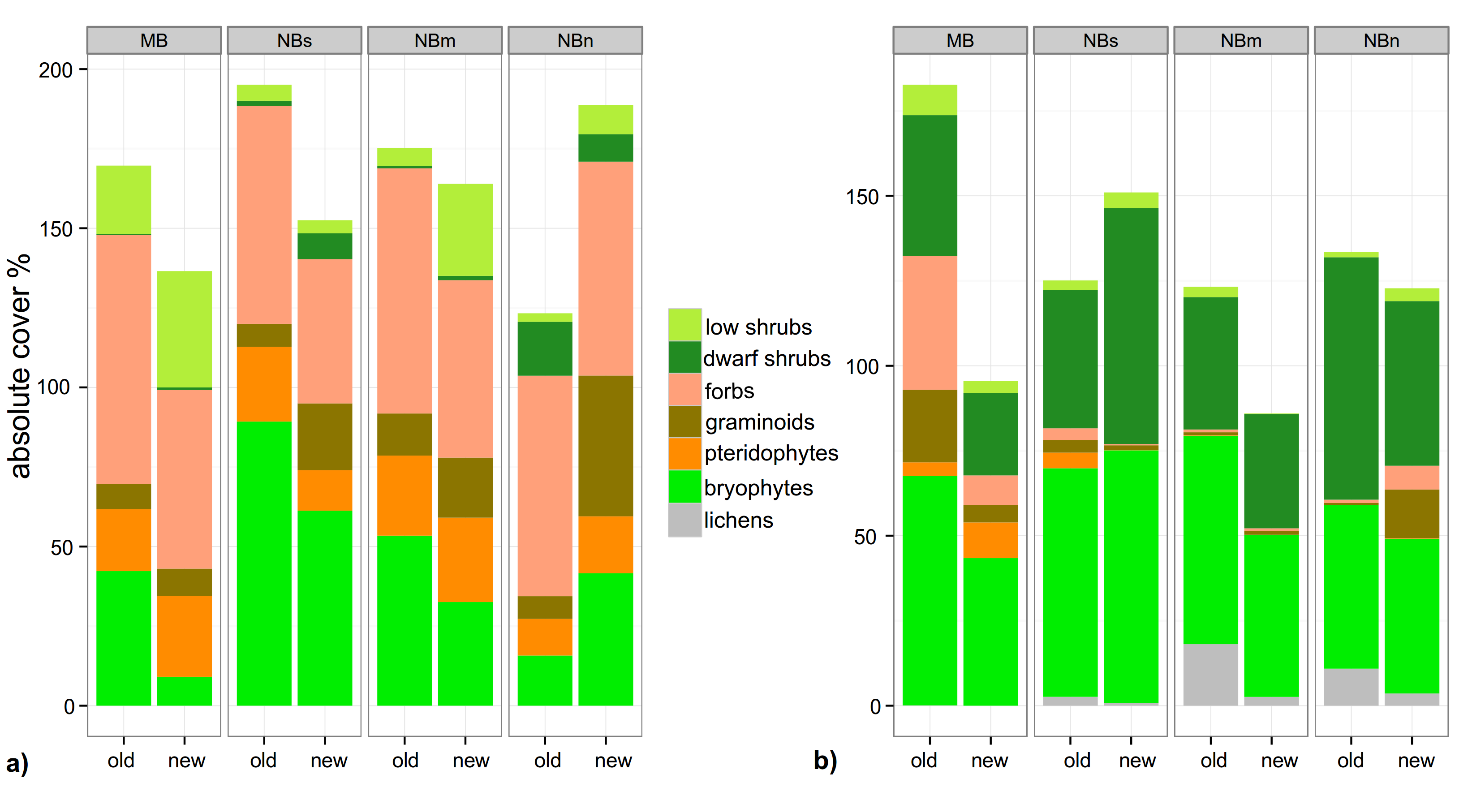


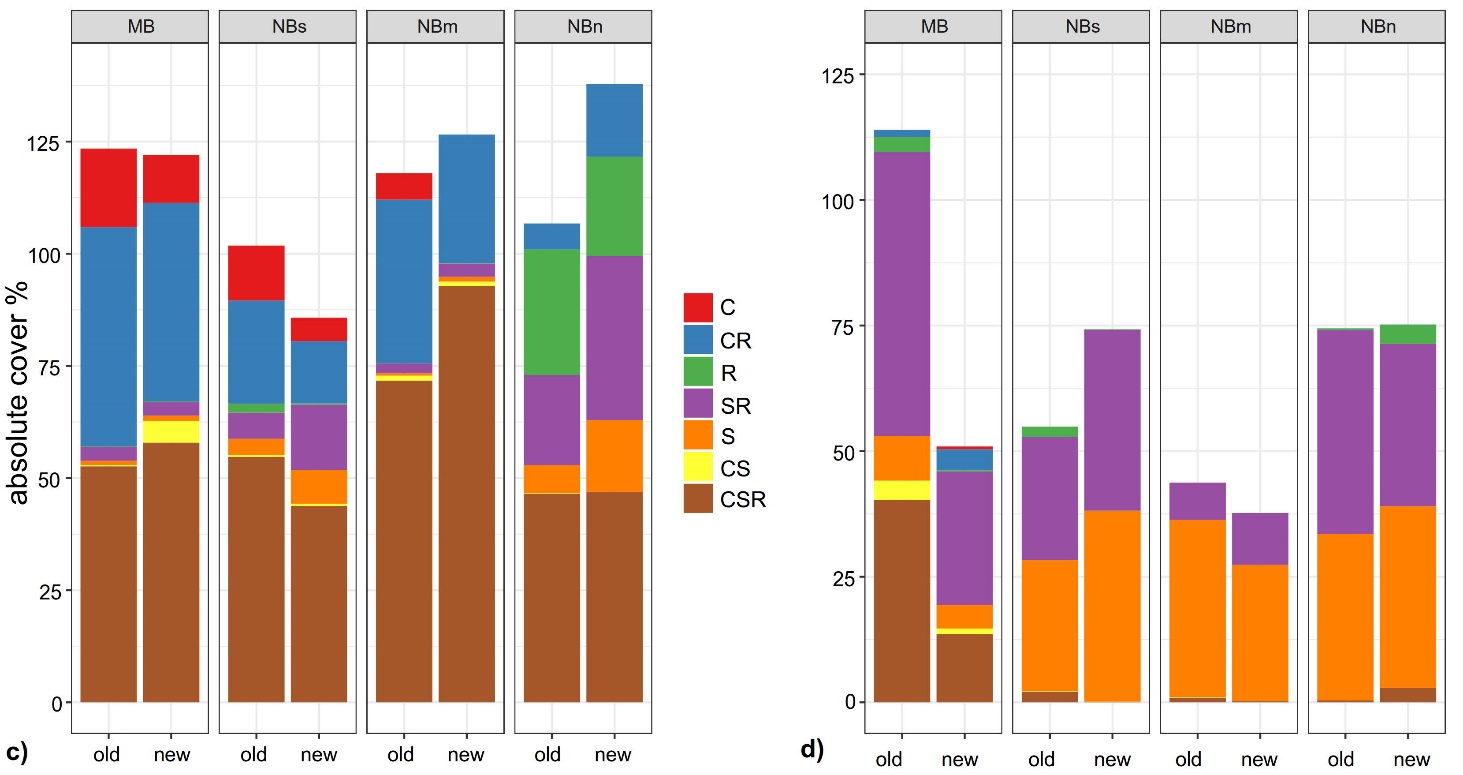


**Appendix S4.** Long-term climate and grazing trends for each studied site. **a)** Thermal sums from 1950 to 2014, **b)** annual precipitation from 1960 to 2014 and **c)** grazing pressure from 1960 to 2013 for each subregion (MB = middle boreal, NBs = northern boreal/southern part, NBm = northern boreal/middle part, NBn = northern boreal/northern part). Thermal sums are reported separately for fertile and infertile sites in subregions NBs and NBm as sampling locations of infertile and fertile sites differed. Loess smoothing function (span 0.4) was used for trend lines. Black dots show the time of original survey and resurvey of each dataset.


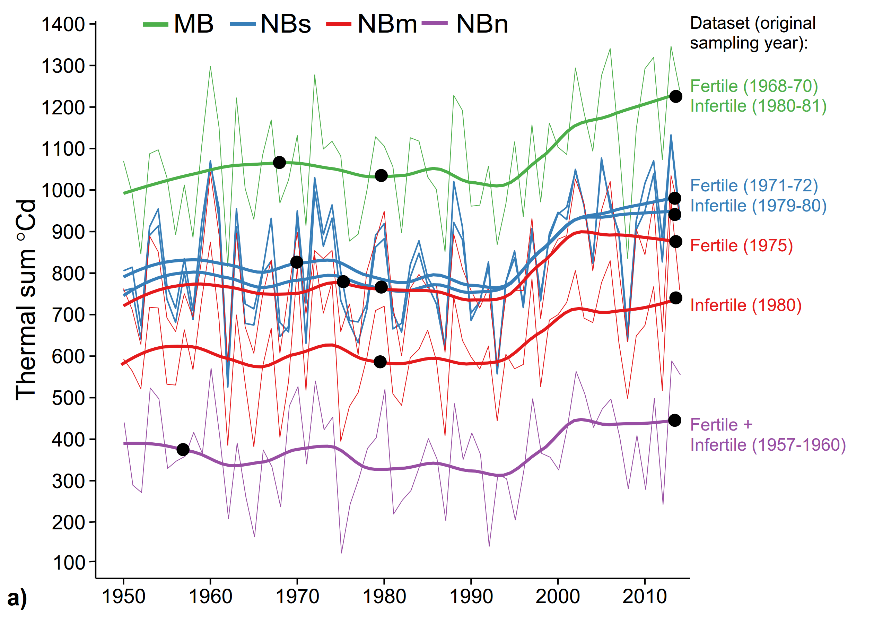

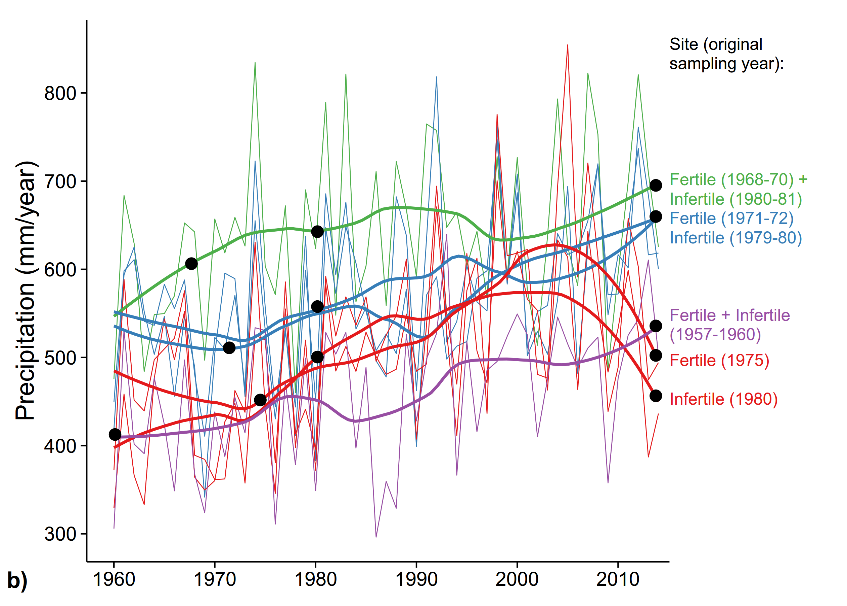


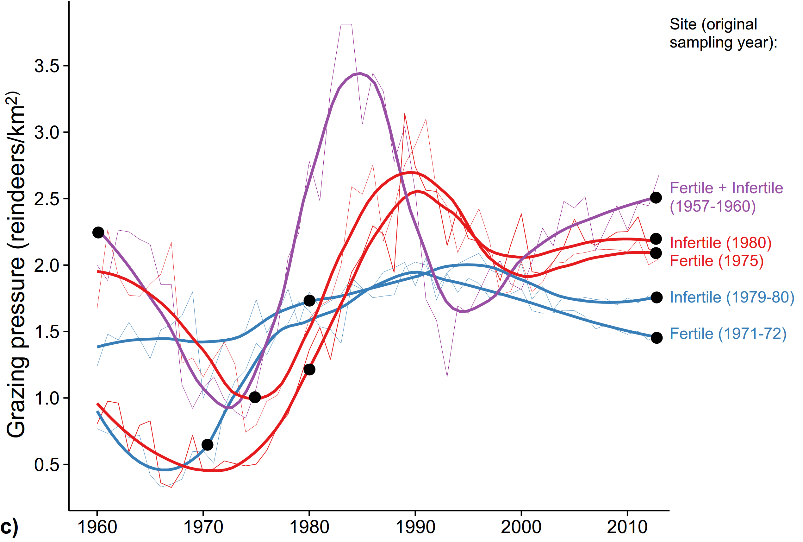


**Appendix S5.** Long-term changes in forest overstoreys. Overlayer estimates (mean ± bootstrapped confidence intervals) for each studied site during original survey (old) and resurvey (new). The variable used for estimating overlayer or canopy cover varied among dataset; **a)** canopy cover scale 0-100 % estimated from 10 x 10 m area, **b)** canopy cover classes 1,2 and 3 for less than 30 %, 30-70 % and more than 70 %, respectively, estimated from 10 x 10 m, **c)** same as previous, **d)** canopy cover scale 0-100 % estimated from 10 x 10 m area, **e)** basal area m^2^/ha, measured using 65 cm chain and 13 mm aperture in both samplings, **f)** canopy cover scale 0-100 % estimated from area of 10 acres **g)**, canopy cover scale 0-100 % estimated from area of 6 acres only during resurvey and **h)** canopy cover scale 0-100 % estimated from 10 x 10 m area.

**
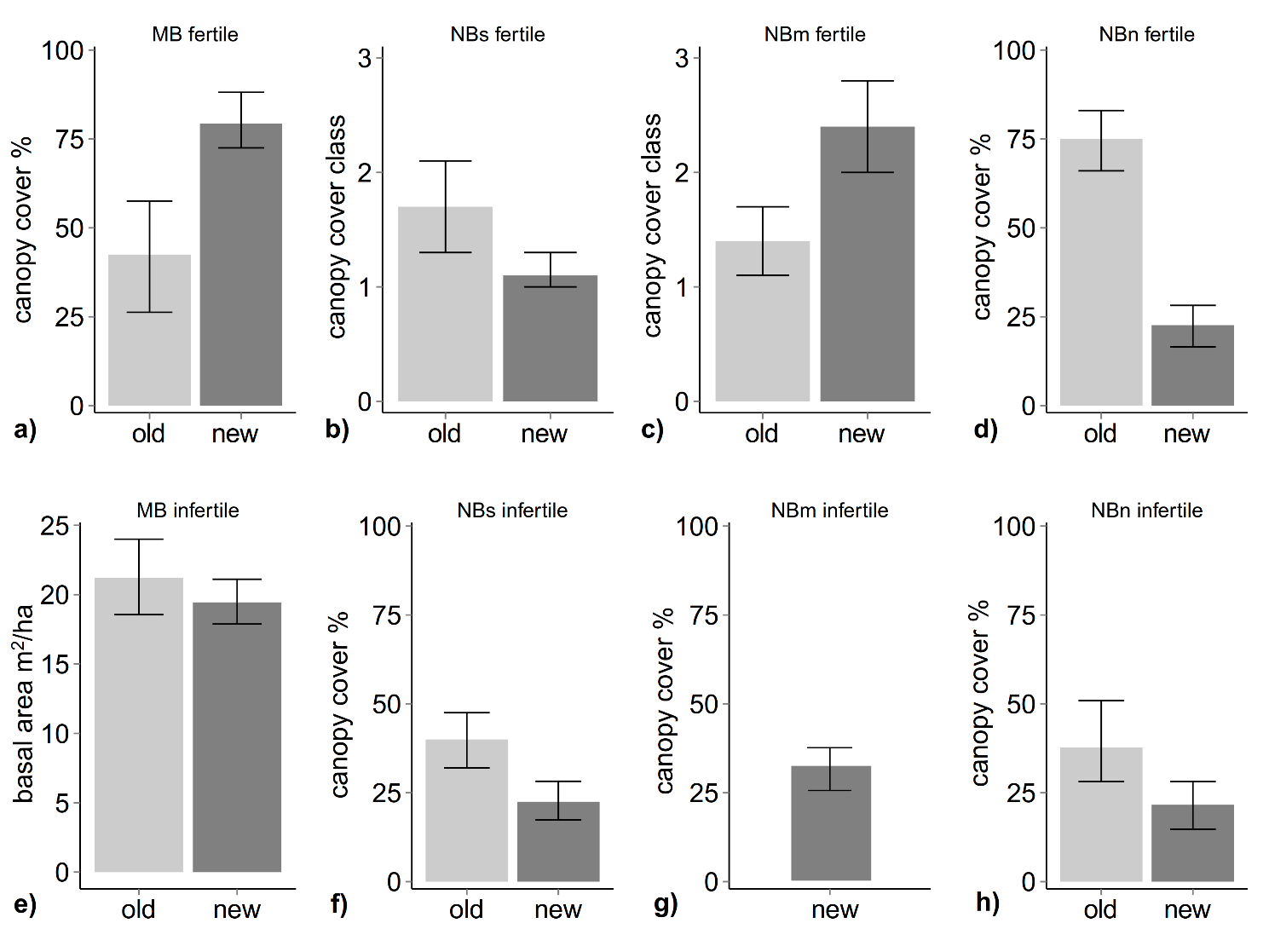
**

**Appendix S6.** The effect of sampling time on the magnitude of temporal turnover.

Five Bayesian models were built to test if the sampling time (i.e. the period between original survey and resurvey) influenced the magnitude of temporal turnover. Model 1 included only the effect of site fertility, in model 2 the linear effect sampling time was added as a covariate, model 3 included the interaction between site fertility and sampling time, in model 4 non-linear effect of sampling time was added and model 5 included non-linear interaction of sampling time and site fertility.

Models were compared using approximate leave-one out cross-validation (function loo_compare from brms package). Models 1 and 3 had equal predictive performance, judged by Expected Log Predictive Density (ELPD). By parsimony, model 1 was selected instead of the more complex model 3.

|  |  | elp_diff | se_diff |
| --- | --- | --- | --- |
| Model 1 | turnover ~ fertility | 0.0 | 0.0 |
| Model 3 | turnover ~ fertility * sampling time | 0.0 | 1.4 |
| Model 5 | turnover ~ fertility + s(sampling time, fertility, bs="fs", k=6) | -0.3 | 1.1 |
| Model 2 | turnover ~ fertility + sampling time | -0.5 | 0.9 |
| Model 4 | turnover ~ fertility + s(sampling time, k=6) | -1.0 | 0.9 |

**Appendix S7.** Fertile herb-rich forest species’ absolute frequencies and mean covers in the original sampling (old) and resampling (new) for the studied sites (MB = middle boreal, NBs = southern part of northern boreal, NBm = middle part of northern boreal and NBn = northern part of northern boreal zone). Only species with frequency ≥ 4 in each subregion are shown. Significant (*p* < 0.05) increases and decreases in frequencies are assessed using chi-squared tests and in mean covers using t-tests for independent samples. Only species with > 3 observations on either of samplings are listed. ‘Sl.’ for shrub layer species.

|  | MB (n=8) | | | | |  | NBs (n=10) | | | | |  | NBm (n=10) | | | | |  | NBn (n=10) | | | | |
| --- | --- | --- | --- | --- | --- | --- | --- | --- | --- | --- | --- | --- | --- | --- | --- | --- | --- | --- | --- | --- | --- | --- | --- |
|  | frequency | |  | mean cover | |  | frequency | |  | mean cover | |  | frequency | |  | mean cover | |  | frequency | |  | mean cover | |
|  | old/new | *p* |  | old/new | *p* |  | old/new | *p* |  | old/new | *p* |  | old/new | *p* |  | old/new | *p* |  | old/new | *p* |  | old/new | *p* |
| **Vascular plants** |  |  |  |  |  |  |  |  |  |  |  |  |  |  |  |  |  |  |  |  |  |  |  |
| *Actaea erythrocarpa* |  |  |  |  |  |  |  |  |  |  |  |  | 4 / 4 | . |  | 0.6 / 1.0 | 0.425 |  |  |  |  |  |  |
| *Actaea spicata* | 2 / 4 | 0.302 |  | 2.1 / 1.0 | 0.574 |  |  |  |  |  |  |  |  |  |  |  |  |  |  |  |  |  |  |
| *Agrostis* spp. |  |  |  |  |  |  | **0 / 8** | **<0.001** |  | **0.0 / 1.3** | **0.026** |  |  |  |  |  |  |  |  |  |  |  |  |
| *Alchemilla* spp. | 6 / 3 | 0.131 |  | 1.1 / 0.2 | 0.181 |  |  |  |  |  |  |  |  |  |  |  |  |  | 5 / 6 | 0.653 |  | 3.2 / 0.5 | 0.197 |
| *Alnus incana,* sl. | **3 / 7** | **0.039** |  | 0.6 / 2.7 | 0.054 |  |  |  |  |  |  |  |  |  |  |  |  |  |  |  |  |  |  |
| *Angelica sylvestris* | 5 / 3 | 0.317 |  | 0.9 / 0.5 | 0.469 |  | 2 / 5 | 0.160 |  | 0.1 / 0.3 | 0.272 |  |  |  |  |  |  |  |  |  |  |  |  |
| *Anthoxanthum alpinum* |  |  |  |  |  |  |  |  |  |  |  |  |  |  |  |  |  |  | 8 / 7 | 0.606 |  | 2.0 / 4.8 | 0.210 |
| *Anthriscus sylvestris* | 6 / 5 | 0.590 |  | 2.5 / 2.3 | 0.901 |  |  |  |  |  |  |  | 2 / 4 | 0.329 |  | 0.3 / 0.7 | 0.418 |  | 4 / 2 | 0.329 |  | 0.8 / 1.2 | 0.701 |
| *Astragalus alpinus* |  |  |  |  |  |  |  |  |  |  |  |  |  |  |  |  |  |  | 6 / 9 | 0.121 |  | 4.2 / 2.0 | 0.197 |
| *Astragalus frigidus* |  |  |  |  |  |  |  |  |  |  |  |  |  |  |  |  |  |  | 4 / 2 | 0.329 |  | 4.2 / 0.2 | 0.099 |
| *Bartsia alpina* |  |  |  |  |  |  |  |  |  |  |  |  |  |  |  |  |  |  | 3 / 4 | 0.639 |  | 0.1 / 0.1 | 0.696 |
| *Betula pubescens*, sl. |  |  |  |  |  |  | 4 / 1 | 0.121 |  | 0.2 / 0.1 | 0.156 |  | 4 / 4 | . |  | 0.3 / 0.9 | 0.247 |  |  |  |  |  |  |
| *Bistorta vivipara* |  |  |  |  |  |  |  |  |  |  |  |  |  |  |  |  |  |  | **3 / 8** | **0.025** |  | 0.1 / 0.7 | 0.242 |
| *Calamagrostis lapponica* |  |  |  |  |  |  |  |  |  |  |  |  |  |  |  |  |  |  | **0 / 6** | **0.003** |  | 0.0 / 0.4 | 0.099 |
| *Calamagrostis purpurea* |  |  |  |  |  |  |  |  |  |  |  |  | **4 / 9** | **0.019** |  | **0.6 / 9.8** | **0.010** |  |  |  |  |  |  |
| *Carex vaginata* |  |  |  |  |  |  |  |  |  |  |  |  |  |  |  |  |  |  | 5 / 9 | 0.051 |  | 0.2 / 1.2 | 0.160 |
| *Cicerbita alpina* |  |  |  |  |  |  | 4 / 4 | . |  | 2.0 / 0.8 | 0.285 |  | 4 / 1 | 0.121 |  | 8.1 / 2.5 | 0.307 |  |  |  |  |  |  |
| *Cirsium helenioides* | 4 / 3 | 0.614 |  | 0.9 / 0.6 | 0.634 |  | 5 / 4 | 0.653 |  | 0.8 / 3.2 | 0.193 |  |  |  |  |  |  |  | 7 / 10 | 0.060 |  | 2.8 / 5.6 | 0.259 |
| *Coeloglossum viride* |  |  |  |  |  |  |  |  |  |  |  |  |  |  |  |  |  |  | 1 / 5 | 0.051 |  | 0.1 / 0.3 | 0.079 |
| *Convallaria majalis* | 6 / 7 | 0.522 |  | 1.5 / 7.9 | 0.050 |  |  |  |  |  |  |  |  |  |  |  |  |  |  |  |  |  |  |
| *Cornus suecica* |  |  |  |  |  |  |  |  |  |  |  |  |  |  |  |  |  |  | 4 / 5 | 0.653 |  | 6.0 / 5.0 | 0.843 |
| *Crepis paludosa* | 4 / 2 | 0.302 |  | 1.1 / 0.8 | 0.708 |  | 5 / 2 | 0.160 |  | 1.2 / 0.1 | 0.297 |  |  |  |  |  |  |  |  |  |  |  |  |
| *Daphne mezereum* |  |  |  |  |  |  | 5 / 4 | 0.653 |  | 0.3 / 0.1 | 0.233 |  |  |  |  |  |  |  |  |  |  |  |  |
| *Deschampsia cespitosa* | 4 / 5 | 0.614 |  | 1.2 / 2.4 | 0.534 |  |  |  |  |  |  |  |  |  |  |  |  |  |  |  |  |  |  |
| *Deschampsia flexuosa* |  |  |  |  |  |  | 4 / 6 | 0.371 |  | 1.5 / 2.4 | 0.501 |  | 3 / 6 | 0.178 |  | 0.6 / 1.4 | 0.301 |  | 10 / 9 | 0.305 |  | **2.9 / 16.6** | **0.012** |
| *Dryopteris carthusiana* | 1 / 4 | 0.106 |  | 0.1 / 0.5 | 0.124 |  |  |  |  |  |  |  |  |  |  |  |  |  |  |  |  |  |  |
| *Dryopteris expansa* | **3 / 7** | **0.039** |  | 0.4 / 7.9 | 0.163 |  |  |  |  |  |  |  | 3 / 6 | 0.178 |  | 9.1 / 13.8 | 0.647 |  |  |  |  |  |  |
| *Elymus caninus* | 3 / 4 | 0.614 |  | 0.5 / 2.5 | 0.310 |  | **1 / 10** | **<0.001** |  | **1.0 / 4.8** | **0.017** |  |  |  |  |  |  |  |  |  |  |  |  |
| *Empetrum hermaphroditum* |  |  |  |  |  |  |  |  |  |  |  |  |  |  |  |  |  |  | 4 / 6 | 0.371 |  | 3.3 / 0.9 | 0.291 |
| *Epilobium angustifolium* |  |  |  |  |  |  |  |  |  |  |  |  | **10 / 5** | **0.010** |  | **4.4 / 0.5** | **0.025** |  | 8 / 5 | 0.160 |  | **0.9 / 0.2** | **0.013** |
| *Equisetum pratense* | 2 / 5 | 0.131 |  | 0.4 / 1.3 | 0.145 |  | 4 / 4 | . |  | 0.9 / 0.1 | 0.181 |  | 7 / 4 | 0.178 |  | 2.3 / 0.8 | 0.243 |  | 6 / 9 | 0.121 |  | **0.3 / 7.9** | **0.041** |
| *Equisetum scirpoides* |  |  |  |  |  |  |  |  |  |  |  |  |  |  |  |  |  |  | 1 / 4 | 0.121 |  | 0.1 / 0.2 | 0.161 |
| *Filipendula ulmaria* | 6 / 5 | 0.590 |  | 14.4 / 8.6 | 0.335 |  | 8 / 9 | 0.531 |  | 12.1 / 3.0 | 0.100 |  |  |  |  |  |  |  |  |  |  |  |  |
| *Festuca ovina* |  |  |  |  |  |  |  |  |  |  |  |  |  |  |  |  |  |  | 3 / 7 | 0.074 |  | **0.2 / 4.6** | **0.021** |
| *Fragaria vesca* | 6 / 3 | 0.131 |  | 1.6 / 0.3 | 0.084 |  | 2 / 4 | 0.329 |  | 0.2 / 0.5 | 0.285 |  |  |  |  |  |  |  |  |  |  |  |  |
| *Galium triflorum* |  |  |  |  |  |  |  |  |  |  |  |  | 2 / 4 | 0.329 |  | 0.3 / 1.3 | 0.172 |  |  |  |  |  |  |
| *Geranium sylvaticum* | 7 / 6 | 0.522 |  | 10.0 / 6.5 | 0.444 |  | 10 / 10 | . |  | 28.6 / 14.9 | 0.084 |  | 10 / 10 | . |  | **36.0 / 19.8** | **0.024** |  | 9 / 10 | 0.305 |  | 17.0 / 7.1 | 0.062 |
| *Geum rivale* |  |  |  |  |  |  | **7 / 0** | **<0.001** |  | 1.1 / 0.0 | 0.066 |  |  |  |  |  |  |  |  |  |  |  |  |
| *Gymnocarpium dryopteris* | 5 / 7 | 0.248 |  | 8.3 / 3.4 | 0.540 |  | 10 / 10 | . |  | 10.4 / 4.5 | 0.201 |  | 9 / 7 | 0.264 |  | 8.4 / 11.3 | 0.567 |  | 7 /9 | 0.264 |  | 10.6 / 9.1 | 0.724 |
| *Hieracium spp.* |  |  |  |  |  |  | 8 / 8 | . |  | 1.4 / 0.8 | 0.158 |  | 5 / 3 | 0.361 |  | 0.6 / 0.5 | 0.890 |  | 3 / 5 | 0.361 |  | 0.1 / 0.3 | 0.165 |
| *Juniperus communis*, sl. |  |  |  |  |  |  | **8 / 1** | **0.002** |  | 0.6 / 0.4 | 0.630 |  |  |  |  |  |  |  | 8 / 10 | 0.136 |  | **1.5 / 8.6** | **0.048** |
| *Linnaea borealis* |  |  |  |  |  |  | 8 / 10 | 0.136 |  | **1.4 / 5.5** | **0.007** |  | 6 / 7 | 0.639 |  | 0.5 / 1.0 | 0.191 |  | 5 / 9 | 0.051 |  | 0.3 / 3.6 | 0.051 |
| *Luzula pilosa* |  |  |  |  |  |  | 9 / 6 | 0.121 |  | **0.9 / 0.2** | **0.018** |  | 6 / 2 | 0.068 |  | **0.4 / 0.1** | **0.037** |  | 8 / 8 | . |  | 0.5 / 1.6 | 0.284 |
| *Lycopodium annotinum* |  |  |  |  |  |  |  |  |  |  |  |  |  |  |  |  |  |  | 5 / 5 | . |  | 0.4 / 0.4 | 0.969 |
| *Maianthemum bifolium* | 8 / 8 | . |  | 4.4 / 3.5 | 0.594 |  | 8 / 10 | 0.136 |  | 2.5 / 1.8 | 0.499 |  | 10 / 7 | 0.060 |  | 2.1 / 1.0 | 0.062 |  |  |  |  |  |  |
| *Melampyrum sylvaticum* |  |  |  |  |  |  | 8 / 7 | 0.606 |  | **1.5 / 0.3** | **0.001** |  |  |  |  |  |  |  | 7 / 8 | 0.606 |  | 1.5 / 0.3 | 0.088 |
| *Melica nutans* |  |  |  |  |  |  | 10 / 10 | . |  | **2.1 / 12.0** | **0.021** |  |  |  |  |  |  |  | 5 / 4 | 0.653 |  | 0.4 / 2.4 | 0.209 |
| *Milium effusum* | 6 / 8 | 0.131 |  | 3.0 / 2.7 | 0.810 |  | 6 / 3 | 0.178 |  | 1.3 / 0.1 | 0.258 |  | 10 / 7 | 0.060 |  | 11.3 / 7.3 | 0.314 |  | 5 / 4 | 0.653 |  | 0.3 / 3.5 | 0.165 |
| *Moneses uniflora* |  |  |  |  |  |  | 5 / 5 | . |  | 1.1 / 0.2 | 0.067 |  |  |  |  |  |  |  |  |  |  |  |  |
| *Myosotis decumbens* |  |  |  |  |  |  |  |  |  |  |  |  |  |  |  |  |  |  | 3 / 5 | 0.361 |  | 0.1 / 0.3 | 0.292 |
| *Orthilia secunda* |  |  |  |  |  |  | 10 / 10 | . |  | 1.8 / 2.2 | 0.541 |  | 3 / 4 | 0.639 |  | 0.2 / 0.2 | 1.000 |  | 1 / 5 | 0.051 |  | 0.1 / 0.4 | 0.139 |
| *Oxalis acetosella* | 8 / 8 | . |  | **14.4 / 5.0** | **0.029** |  |  |  |  |  |  |  |  |  |  |  |  |  |  |  |  |  |  |
| *Paris quadrifolia* | 7 / 8 | 0.302 |  | 8.5 / 2.4 | 0.247 |  | 7 / 6 | 0.639 |  | 1.7 / 0.3 | 0.193 |  | 6 / 9 | 0.121 |  | 1.2 / 1.3 | 0.800 |  |  |  |  |  |  |
| *Pedicularis lapponica* |  |  |  |  |  |  |  |  |  |  |  |  |  |  |  |  |  |  | 6 / 4 | 0.371 |  | 0.2 / 0.2 | 0.750 |
| *Phleum alpinum* |  |  |  |  |  |  |  |  |  |  |  |  |  |  |  |  |  |  | **0 / 6** | **0.003** |  | 0.0 / 0.2 | 0.057 |
| *Picea abies*, sl. | 2 / 5 | 0.131 |  | 0.3 / 0.9 | 0.149 |  | 5 / 6 | 0.653 |  | 0.2 / 0.9 | 0.104 |  |  |  |  |  |  |  |  |  |  |  |  |
| *Poa alpigena* |  |  |  |  |  |  |  |  |  |  |  |  |  |  |  |  |  |  | 3 / 7 | 0.074 |  | **0.1 / 5.5** | **0.040** |
| *Poa nemoralis* |  |  |  |  |  |  |  |  |  |  |  |  | 1 / 4 | 0.121 |  | 0.1 / 0.3 | 0.247 |  | 4 / 7 | 0.178 |  | 0.1 / 2.7 | 0.126 |
| *Prunella vulgaris* | **0 / 4** | **0.021** |  | 0.0 / 0.4 | 0.051 |  |  |  |  |  |  |  |  |  |  |  |  |  |  |  |  |  |  |
| *Prunus padus, sl.* | 7 / 8 | 0.302 |  | 12.1 / 21.4 | 0.346 |  |  |  |  |  |  |  | 8 / 7 | 0.606 |  | **2.2 / 20.0** | **0.015** |  |  |  |  |  |  |
| *Pyrola minor* |  |  |  |  |  |  | 5 / 5 | . |  | 0.4 / 0.3 | 0.879 |  |  |  |  |  |  |  | 5 / 4 | 0.653 |  | 0.2 / 0.3 | 0.558 |
| *Ranunculus acris* | 5 / 1 | 0.094 |  | 1.1 / 0.1 | 0.168 |  | 8 / 7 | 0.606 |  | 0.7 / 0.4 | 0.254 |  |  |  |  |  |  |  | 4 / 8 | 0.068 |  | 0.5 / 4.6 | **0.048** |
| *Ranunculus repens* |  |  |  |  |  |  |  |  |  |  |  |  | **0 / 4** | **0.025** |  | 0.0 / 0.3 | 0.061 |  |  |  |  |  |  |
| *Ribes spicatum*, sl. | 4 / 6 | 0.302 |  | 2.3 / 0.5 | 0.208 |  | 5 / 9 | 0.051 |  | **0.3 / 1.4** | **0.025** |  | 6 / 9 | 0.121 |  | 1.8 / 6.5 | 0.078 |  |  |  |  |  |  |
| *Rubus idaeus*, sl. | 4 / 7 | 0.106 |  | 4.0 / 6.5 | 0.602 |  |  |  |  |  |  |  |  |  |  |  |  |  |  |  |  |  |  |
| *Rubus saxatilis* | 4 / 4 | . |  | 1.1 / 1.3 | 0.890 |  | 8 / 9 | 0.531 |  | **1.6 / 4.2** | **0.043** |  | 9 / 10 | 0.305 |  | 7.9 / 9.8 | 0.586 |  | 9 / 9 | . |  | 3.9 / 3.3 | 0.747 |
| *Rumex* ssp. |  |  |  |  |  |  |  |  |  |  |  |  |  |  |  |  |  |  | **0 / 6** | **0.003** |  | 0.0 / 0.2 | 0.074 |
| *Salix myrsinifolia ssp.borealis* | |  |  |  |  |  |  |  |  |  |  |  |  |  |  |  |  |  | **7 / 2** | **0.025** |  | 0.5 / 0.4 | 0.733 |
| *Saussurea alpina* |  |  |  |  |  |  |  |  |  |  |  |  |  |  |  |  |  |  | **1 / 8** | **0.002** |  | **0.1 / 2.9** | **0.048** |
| *Selaginella selaginoides* |  |  |  |  |  |  | 5 / 8 | 0.068 |  | 0.4 / 0.3 | 0.614 |  |  |  |  |  |  |  | 5 / 4 | 0.653 |  | 0.1 / 0.1 | 0.943 |
| *Silene dioica* | **0 / 7** | **<0.001** |  | 0.0 / 3.5 | 0.079 |  |  |  |  |  |  |  |  |  |  |  |  |  | 6 / 9 | 0.121 |  | 0.4 / 0.6 | 0.584 |
| *Solidago virgaurea* |  |  |  |  |  |  | 7 / 9 | 0.264 |  | 1.4 / 2.1 | 0.288 |  | 7 / 3 | 0.074 |  | 1.3 / 0.4 | 0.077 |  | 9 / 10 | 0.305 |  | **1.2 / 3.5** | **0.035** |
| *Sorbus aucuparia*, sl. | **3 / 8** | **0.007** |  | **0.3 / 4.3** | **0.004** |  | 6 / 7 | 0.639 |  | 0.3 / 0.5 | 0.580 |  | 9 / 6 | 0.121 |  | 1.1 / 1.0 | 0.839 |  |  |  |  |  |  |
| *Stellaria nemorum* |  |  |  |  |  |  |  |  |  |  |  |  | 6 / 6 | . |  | 5.6 / 14.0 | 0.140 |  |  |  |  |  |  |
| *Taraxacum spp.* |  |  |  |  |  |  | 4 / 2 | 0.329 |  | 0.1 / 0.4 | 0.466 |  |  |  |  |  |  |  | 4 / 3 | 0.639 |  | 0.1 / 0.1 | 0.940 |
| *Trientalis europaea* | 7 / 6 | 0.522 |  | 2.7 / 0.7 | 0.291 |  | 10 / 10 | . |  | 1.2 / 0.6 | 0.092 |  | 8 / 6 | 0.329 |  | 0.9 / 0.3 | 0.083 |  | 6 / 7 | 0.639 |  | 0.3 / 0.4 | 0.495 |
| *Trifolium repens* |  |  |  |  |  |  | 3 / 5 | 0.361 |  | 0.6 / 0.9 | 0.706 |  |  |  |  |  |  |  |  |  |  |  |  |
| *Trollius europaea* | 4 / 4 | . |  | 2.1 / 1.6 | 0.680 |  |  |  |  |  |  |  |  |  |  |  |  |  | 6 / 9 | 0.121 |  | 6.6 / 12.2 | 0.275 |
| *Vaccinium myrtillus* |  |  |  |  |  |  | 4 / 8 | 0.068 |  | 0.7 / 4.1 | 0.195 |  | 4 / 4 | . |  | 0.1 / 0.3 | 0.343 |  | 9 / 8 | 0.531 |  | 12.2 / 1.3 | 0.149 |
| *Vaccinium uliginosum* |  |  |  |  |  |  |  |  |  |  |  |  |  |  |  |  |  |  | 4 / 3 | 0.639 |  | 0.4 / 0.6 | 0.814 |
| *Vaccinium vitis-idaea* |  |  |  |  |  |  | 7 / 10 | 0.060 |  | **1.0 / 4.0** | **0.017** |  | 4 / 5 | 0.653 |  | 0.3 / 0.4 | 0.481 |  | 8 / 10 | 0.136 |  | **0.8 / 5.8** | **0.022** |
| *Viola biflora* |  |  |  |  |  |  |  |  |  |  |  |  |  |  |  |  |  |  | 9 / 10 | 0.305 |  | 16.0 / 9.4 | 0.328 |
| *Viola epipsila* |  |  |  |  |  |  | **6 / 1** | **0.019** |  | **2.9 / 0.1** | **0.026** |  |  |  |  |  |  |  |  |  |  |  |  |
| *Viola mirabilis* | **6 / 2** | **0.046** |  | 2.5 / 4.4 | 0.636 |  |  |  |  |  |  |  |  |  |  |  |  |  |  |  |  |  |  |
| *Viola selkirkii* | 6 / 4 | 0.302 |  | 1.5 / 0.7 | 0.349 |  |  |  |  |  |  |  | 1 / 4 | 0.121 |  | 0.3 / 0.6 | 0.487 |  |  |  |  |  |  |
| **Bryophytes** |  |  |  |  |  |  |  |  |  |  |  |  |  |  |  |  |  |  |  |  |  |  |  |
| *Barbilophozia lycopodioides* |  |  |  |  |  |  | 6 / 9 | 0.121 |  | 1.7 / 0.6 | 0.116 |  | **2 / 9** | **0.002** |  | **0.1 / 3.6** | **0.003** |  | 6 / 9 | 0.121 |  | 0.4 / 1.7 | 0.067 |
| *Barbilophozia* spp. |  |  |  |  |  |  |  |  |  |  |  |  |  |  |  |  |  |  | 2 / 5 | 0.160 |  | 0.1 / 0.1 | 0.799 |
| *Brachythecium* spp. | 7 / 4 | 0.106 |  | **10.7 / 0.4** | **0.017** |  | 5 / 7 | 0.361 |  | 3.9 / 2.2 | 0.364 |  | 10 / 10 | . |  | **29.3 / 8.9** | **0.026** |  | 8 / 10 | 0.136 |  | 8.1 / 13.8 | 0.271 |
| *Bryum* spp. |  |  |  |  |  |  | **4 / 0** | **0.025** |  | 5.5 / 0.0 | 0.093 |  |  |  |  |  |  |  |  |  |  |  |  |
| *Climacium dendroides* |  |  |  |  |  |  |  |  |  |  |  |  |  |  |  |  |  |  | 3 / 4 | 0.639 |  | 0.1 / 0.2 | 0.151 |
| *Dicranum majus* |  |  |  |  |  |  | **2 / 7** | **0.025** |  | 0.6 / 0.6 | 0.942 |  |  |  |  |  |  |  |  |  |  |  |  |
| *Dicranum scoparium* |  |  |  |  |  |  |  |  |  |  |  |  | **0 / 5** | **0.010** |  | **0.0 / 0.4** | **0.019** |  | 4 / 4 | . |  | 1.1 / 0.8 | 0.786 |
| *Hylocomiastrum pyrenaicum* | **7 / 0** | **<0.001** |  | **16.3 / 0.0** | **0.003** |  | 5 / 2 | 0.160 |  | 9.7 / 0.1 | 0.107 |  |  |  |  |  |  |  |  |  |  |  |  |
| *Hylocomium splendens* |  |  |  |  |  |  | 9 / 9 | . |  | 9.3 / 11.0 | 0.755 |  | 4 / 8 | 0.068 |  | 1.3 / 7.1 | 0.128 |  | 6 / 7 | 0.639 |  | 2.2 / 4.1 | 0.410 |
| *Mnium spinosum* |  |  |  |  |  |  |  |  |  |  |  |  |  |  |  |  |  |  | 6 / 8 | 0.329 |  | 0.3 / 1.5 | 0.244 |
| *Mnium stellare* |  |  |  |  |  |  |  |  |  |  |  |  | 4 / 1 | 0.121 |  | 0.7 / 0.1 | 0.230 |  |  |  |  |  |  |
| *Plagiomnium cuspidatum* | **2 / 6** | **0.046** |  | 2.5 / 0.5 | 0.270 |  |  |  |  |  |  |  |  |  |  |  |  |  |  |  |  |  |  |
| *Plagiomnium ellipticum* |  |  |  |  |  |  | 5 / 3 | 0.361 |  | 11.2 / 0.5 | 0.051 |  | 9 / 8 | 0.531 |  | 14.3 / 4.2 | 0.083 |  |  |  |  |  |  |
| *Pleurozium schreberi* | 6 / 4 | 0.302 |  | 2.8 / 0.8 | 0.193 |  | **2 / 10** | **<0.001** |  | 0.7 / 1.9 | 0.098 |  | **3 / 8** | **0.025** |  | **0.3 / 1.9** | **0.030** |  | **2 / 7** | **0.025** |  | 3.0 / 3.0 | 0.990 |
| *Polytrichum commune* |  |  |  |  |  |  |  |  |  |  |  |  | 2 / 4 | 0.329 |  | 0.1 / 0.4 | 0.404 |  | 3 / 5 | 0.361 |  | 0.1 / 0.6 | 0.387 |
| *Ptilium crista-castrensis* |  |  |  |  |  |  | **0 / 4** | **0.025** |  | 0.0 / 0.7 | 0.126 |  |  |  |  |  |  |  |  |  |  |  |  |
| *Rhodobryum roseum* |  |  |  |  |  |  |  |  |  |  |  |  | 6 / 7 | 0.639 |  | 4.7 / 2.7 | 0.420 |  | **1 / 7** | **0.006** |  | 0.1 / 1.4 | 0.052 |
| *Rhytidiadelphus triquetrus* |  |  |  |  |  |  | 10 / 10 | . |  | 43.5 / 35.6 | 0.542 |  |  |  |  |  |  |  |  |  |  |  |  |
| *Rhytidiadelphus subpinnatus* |  |  |  |  |  |  | 2 / 5 | 0.160 |  | 1.5 / 1.6 | 0.922 |  |  |  |  |  |  |  |  |  |  |  |  |

**Appendix S8.** Infertile heath forest species’ absolute frequencies and mean covers in the original sampling (old) and resampling (new) for the studied sites (MB = middle boreal, NBs = southern part of northern boreal, NBm = middle part of northern boreal and NBn = northern part of northern boreal zone). Only species with frequency ≥ 4 in each subregion are shown. Significant (*p* < 0.05) increases and decreases in frequencies are assessed using chi-squared tests and in mean covers using t-tests for independent samples. ‘Sl.’ for shrub layer species.

|  | MB (n=9) | | | | |  | NBs (n=10) | | | | |  | NBm (n=10) | | | | |  | NBn (n=11) | | | | |
| --- | --- | --- | --- | --- | --- | --- | --- | --- | --- | --- | --- | --- | --- | --- | --- | --- | --- | --- | --- | --- | --- | --- | --- |
|  | frequency | |  | mean cover | |  | frequency | |  | mean cover | |  | frequency | |  | mean cover | |  | frequency | |  | mean cover | |
|  | old/new | *p* |  | old/new | *p* |  | old/new | *p* |  | old/new | *p* |  | old/new | *p* |  | old/new | *p* |  | old/new | *p* |  | old/new | *p* |
| **Vascular plants** |  |  |  |  |  |  |  |  |  |  |  |  |  |  |  |  |  |  |  |  |  |  |  |
| *Agrostis capillaris* | 6 / 3 | 0.157 |  | 0.9 / 0.1 | 0.055 |  |  |  |  |  |  |  |  |  |  |  |  |  |  |  |  |  |  |
| *Andromeda polifolia* |  |  |  |  |  |  | **0 / 7** | **0.001** |  | **0.0 / 0.4** | **0.005** |  |  |  |  |  |  |  |  |  |  |  |  |
| *Arctostaphylos uva-ursi* |  |  |  |  |  |  |  |  |  |  |  |  | 4 / 2 | 0.329 |  | 0.2 / 0.1 | 0.490 |  |  |  |  |  |  |
| *Betula pubescens*, sl. | **8 / 1** | **<0.001** |  | 1.0 / 0.3 | 0.134 |  | **4 / 10** | **0.003** |  | **0.2 / 1.1** | **0.029** |  |  |  |  |  |  |  |  |  |  |  |  |
| *Calluna vulgaris* |  |  |  |  |  |  | **5 / 10** | **0.010** |  | 4.8 / 6.8 | 0.550 |  | 10 / 10 | . |  | 15.2 / 8.6 | 0.128 |  |  |  |  |  |  |
| *Calamagrostis purpurea* | 1 / 4 | 0.106 |  | 0.1 / 0.3 | 0.188 |  |  |  |  |  |  |  |  |  |  |  |  |  |  |  |  |  |  |
| *Carex globularis* |  |  |  |  |  |  | **1 / 6** | **0.019** |  | 0.2 / 0.4 | 0.192 |  |  |  |  |  |  |  |  |  |  |  |  |
| *Cornus suecica* |  |  |  |  |  |  |  |  |  |  |  |  |  |  |  |  |  |  | 3 / 4 | 0.647 |  | 0.3 / 3.4 | 0.288 |
| *Deschampsia cespitosa* | **0 / 4** | **0.021** |  | 0.1 / 0.2 | 0.173 |  |  |  |  |  |  |  |  |  |  |  |  |  |  |  |  |  |  |
| *Deschampsia flexuosa* | 9 / 9 | . |  | 16.6 / 3.9 | 0.097 |  | 9 / 10 | 0.305 |  | **2.6 / 0.9** | **0.017** |  | 7 / 7 | . |  | 0.5 / 0.9 | 0.458 |  | 10 / 11 | 0.306 |  | **0.5 / 14.4** | **<0.001** |
| *Diphasiastrum complanatum* |  |  |  |  |  |  | 5 / 1 | 0.051 |  | 4.4 / 0.1 | 0.057 |  | **4 / 0** | **0.025** |  | **0.1 / 0.0** | **0.037** |  |  |  |  |  |  |
| *Dryopteris carthusiana* | 2 / 4 | 0.302 |  | 0.1 / 0.5 | 0.091 |  |  |  |  |  |  |  |  |  |  |  |  |  |  |  |  |  |  |
| *Empetrum nigrum* |  |  |  |  |  |  | 10 / 10 | . |  | **8.9 / 24.5** | **0.001** |  | 10 / 10 | . |  | 12.9 / 12.0 | 0.821 |  | 11 / 11 | . |  | 30.0 / 24.1 | 0.289 |
| *Geranium sylvaticum* | 5 / 3 | 0.343 |  | 3.0 / 0.9 | 0.270 |  |  |  |  |  |  |  |  |  |  |  |  |  |  |  |  |  |  |
| *Gymnocarpium dryopteris* | 4 / 5 | 0.637 |  | 3.7 / 6.7 | 0.529 |  |  |  |  |  |  |  |  |  |  |  |  |  |  |  |  |  |  |
| *Juniperus communis*, sl. | **7 / 1** | **0.004** |  | 2.2 / 0.1 | 0.100 |  | 9 / 7 | 0.264 |  | 1.7 / 1.5 | 0.867 |  |  |  |  |  |  |  | 7 / 10 | 0.127 |  | 1.0 / 4.0 | 0.121 |
| *Linnaea borealis* | 7 / 6 | 0.599 |  | 2.8 / 0.4 | 0.102 |  | 5 / 1 | 0.051 |  | 0.3 / 0.1 | 0.101 |  | 3 / 5 | 0.361 |  | 0.1 / 0.9 | 0.193 |  | 9 / 9 | . |  | 0.3 / 1.1 | 0.118 |
| *Luzula pilosa* | 8 / 8 | . |  | **2.1 / 0.3** | **0.019** |  | 4 / 1 | 0.121 |  | 0.2 / 0.1 | 0.150 |  |  |  |  |  |  |  | 1 / 4 | 0.127 |  | 0.1 / 0.2 | 0.155 |
| *Maianthemum bifolium* | 9 / 9 | . |  | **16.6 / 3.2** | **0.010** |  | **7 / 1** | **0.006** |  | 0.6 / 0.1 | 0.051 |  |  |  |  |  |  |  |  |  |  |  |  |
| *Melampyrum pratense* | 9 / 6 | 0.058 |  | **3.0 / 0.2** | **0.035** |  | 4 / 1 | 0.121 |  | 0.2 / 0.1 | 0.058 |  |  |  |  |  |  |  | 2 / 5 | 0.170 |  | 0.1 / 0.2 | 0.505 |
| *Melampyrun sylvaticum* | 7 / 5 | 0.317 |  | **2.9 / 0.2** | **0.048** |  | 4 / 5 | 0.653 |  | 0.3 / 0.1 | 0.315 |  |  |  |  |  |  |  |  |  |  |  |  |
| *Orthilia secunda* |  |  |  |  |  |  | **0 / 4** | **0.025** |  | 0.0 / 0.2 | 0.141 |  |  |  |  |  |  |  |  |  |  |  |  |
| *Oxalis acetosella* | 4 / 5 | 0.637 |  | 0.6 / 1.1 | 0.459 |  |  |  |  |  |  |  |  |  |  |  |  |  |  |  |  |  |  |
| *Pedicularis lapponica* |  |  |  |  |  |  |  |  |  |  |  |  |  |  |  |  |  |  | 2 / 6 | 0.076 |  | 0.1 / 0.1 | 0.159 |
| *Picea abies*, sl. | 9 / 7 | 0.134 |  | 1.2 / 1.9 | 0.388 |  | 7 / 10 | 0.060 |  | **0.4 / 0.9** | **0.025** |  |  |  |  |  |  |  |  |  |  |  |  |
| *Pinus sylvestris*, sl. |  |  |  |  |  |  | 4 / 4 | . |  | 0.1 / 0.1 | 0.881 |  | 5 / 4 | 0.653 |  | 0.2 / 0.1 | 0.404 |  |  |  |  |  |  |
| *Populus tremula*, sl. | **4 / 0** | **0.021** |  | 0.3 / 0.0 | 0.173 |  |  |  |  |  |  |  |  |  |  |  |  |  |  |  |  |  |  |
| *Solidago virgaurea* | 6 / 7 | 0.599 |  | **6.4 / 1.3** | **0.026** |  |  |  |  |  |  |  |  |  |  |  |  |  | 8 / 7 | 0.647 |  | 0.1 / 0.5 | 0.169 |
| *Sorbus aucuparia*, sl. | 7 / 8 | 0.527 |  | 3.2 / 0.8 | 0.181 |  |  |  |  |  |  |  |  |  |  |  |  |  |  |  |  |  |  |
| *Trientalis europaea* | 7 / 7 | . |  | 0.9 / 0.4 | 0.123 |  |  |  |  |  |  |  | 4 / 3 | 0.639 |  | 0.2 / 0.1 | 0.502 |  | 5 / 8 | 0.193 |  | 0.1 / 1.5 | 0.069 |
| *Vaccinium myrtillus* | 9 / 9 | . |  | 35.9 / 21.7 | 0.097 |  | 10 / 10 | . |  | 18.6 / 21.4 | 0.619 |  | 10 / 10 | . |  | 5.6 / 6.9 | 0.611 |  | 11 / 11 | . |  | **38.6 / 12.5** | **0.002** |
| *Vaccinium uliginosa* |  |  |  |  |  |  | 9 / 10 | 0.305 |  | **2.9 / 13.6** | **<0.001** |  | 5 / 4 | 0.653 |  | 1.1 / 1.3 | 0.898 |  | 6 / 7 | 0.665 |  | 1.0 / 3.3 | 0.145 |
| *Vaccinium vitis-idaea* | 9 / 9 | . |  | 5.0 / 2.1 | 0.167 |  | 10 / 10 | . |  | **5.1 / 2.5** | **0.005** |  | 10 / 10 | . |  | **3.8 / 6.0** | **0.028** |  | 11 / 11 | . |  | **1.2 / 7.7** | **0.009** |
| **Bryophytes** |  |  |  |  |  |  |  |  |  |  |  |  |  |  |  |  |  |  |  |  |  |  |  |
| *Brachythecium* spp. | 8 / 7 | 0.527 |  | 1.2 / 0.5 | 0.366 |  |  |  |  |  |  |  |  |  |  |  |  |  | **0 / 4** | **0.027** |  | **0.0 / 0.1** | **0.038** |
| *Barbilophozia lycopodioides* | 8 / 7 | 0.527 |  | 8.9 / 3.8 | 0.212 |  | **5 / 10** | **0.010** |  | 4.1 / 7.6 | 0.383 |  | 10 / 8 | 0.136 |  | 1.9 / 2.6 | 0.558 |  | **6 / 11** | **0.011** |  | 2.8 / 3.5 | 0.749 |
| *Barbilophozia* spp. | **0 / 4** | **0.021** |  | 0.0 / 0.2 | 0.109 |  | 6 / 9 | 0.121 |  | 6.1 / 3.9 | 0.569 |  | 6 / 8 | 0.329 |  | **0.3 / 1.3** | **0.023** |  | 6 / 9 | 0.170 |  | 2.0 / 1.2 | 0.548 |
| *Dicranum drummondii* |  |  |  |  |  |  | 4 / 4 | . |  | 1.4 / 0.3 | 0.170 |  | 4 / 7 | 0.178 |  | **0.3 / 1.4** | **0.012** |  |  |  |  |  |  |
| *Dicranum fuscescens* | 4 / 2 | 0.302 |  | 0.6 / 0.2 | 0.387 |  | 8 / 10 | 0.136 |  | 4.2 / 2.1 | 0.217 |  | 8 / 9 | 0.531 |  | **19.7 / 3.8** | **0.044** |  | 5 / 7 | 0.392 |  | 1.8 / 1.3 | 0.662 |
| *Dicranum majus* | 7 / 8 | 0.527 |  | 3.2 / 3.2 | 0.995 |  | 6 / 8 | 0.329 |  | 1.6 / 2.4 | 0.527 |  |  |  |  |  |  |  |  |  |  |  |  |
| *Dicranum polysetum* | 7 / 5 | 0.317 |  | 0.6 / 1.0 | 0.719 |  | 2 / 4 | 0.329 |  | 0.1 / 0.5 | 0.132 |  | 6 / 5 | 0.653 |  | 0.6 / 0.7 | 0.887 |  |  |  |  |  |  |
| *Dicranum scoparium* | 9 / 6 | 0.058 |  | **7.0 / 1.3** | **0.040** |  | **0 / 9** | **<0.001** |  | **0.0 / 2.9** | **0.003** |  | 9 / 10 | 0.305 |  | **1.0 / 3.9** | **0.024** |  | 8 / 11 | 0.062 |  | 11.2 / 4.0 | 0.074 |
| *Hylocomium splendens* | 9 / 8 | 0.303 |  | 14.7 / 8.0 | 0.397 |  | 8 / 10 | 0.136 |  | 10.0 / 9.0 | 0.825 |  | 4 / 6 | 0.371 |  | 4.2 / 4.9 | 0.858 |  | 5 / 9 | 0.076 |  | 1.8 / 6.5 | 0.185 |
| *Pleurozium schreberi* | 9 / 9 | . |  | 18.5 / 12.7 | 0.453 |  | 10 / 10 | . |  | 30.5 / 16.7 | 0.110 |  | 10 / 10 | . |  | 29.6 / 24.1 | 0.512 |  | 10 / 11 | 0.306 |  | 25.9 / 28.6 | 0.720 |
| *Polytrichum commune* | 5 / 5 | . |  | 1.5 / 2.4 | 0.609 |  | **2 / 10** | **<0.001** |  | **0.6 / 5.9** | **0.046** |  |  |  |  |  |  |  | **1 / 7** | **0.008** |  | 0.3 / 0.9 | 0.233 |
| *Polytrichum juniperinum* | **5 / 1** | **0.046** |  | 0.1 / 0.0 | 0.051 |  |  |  |  |  |  |  | 5 / 1 | 0.051 |  | 2.4 / 0.1 | 0.322 |  | 7 / 10 | 0.127 |  | 2.1 / 1.5 | 0.560 |
| *Polytrichum strictum* |  |  |  |  |  |  | **0 / 6** | **0.003** |  | 0.0 / 1.3 | 0.063 |  |  |  |  |  |  |  |  |  |  |  |  |
| *Ptilium crista-castrensis* |  |  |  |  |  |  | 4 / 4 | . |  | 6.5 / 0.3 | 0.209 |  |  |  |  |  |  |  |  |  |  |  |  |
| *Rhodobryum roseum* | 4 / 5 | 0.637 |  | 0.4 / 0.1 | 0.395 |  |  |  |  |  |  |  |  |  |  |  |  |  |  |  |  |  |  |
| *Rhytidiadelphus triquetrus* | 7 / 5 | 0.317 |  | 6.7 / 4.3 | 0.540 |  |  |  |  |  |  |  |  |  |  |  |  |  |  |  |  |  |  |
| *Sphagnum* spp. |  |  |  |  |  |  | **1 / 7** | **0.006** |  | **0.3 / 21.0** | **0.015** |  |  |  |  |  |  |  |  |  |  |  |  |
| **Lichens** |  |  |  |  |  |  |  |  |  |  |  |  |  |  |  |  |  |  |  |  |  |  |  |
| *Cladonia arbuscula* |  |  |  |  |  |  | 7 / 3 | 0.074 |  | 0.6 / 0.2 | 0.096 |  | 9 / 9 | . |  | **7.5 / 0.7** | **0.045** |  | 11 / 10 | 0.306 |  | 1.4 / 0.7 | 0.319 |
| *Cladonia bellidiflora* |  |  |  |  |  |  | 4 / 2 | 0.329 |  | 0.1 / 0.1 | 0.168 |  |  |  |  |  |  |  |  |  |  |  |  |
| *Cladonia crispata* |  |  |  |  |  |  |  |  |  |  |  |  | 8 / 7 | 0.606 |  | **0.4 / 0.1** | **0.018** |  | **7 / 2** | **0.030** |  | 0.2 / 0.1 | 0.173 |
| *Cladonia rangiferina* |  |  |  |  |  |  | 4 / 6 | 0.371 |  | 0.3 / 0.2 | 0.846 |  | 10 / 9 | 0.305 |  | **4.6 / 0.8** | **0.045** |  | 9 / 10 | 0.534 |  | 0.6 / 0.5 | 0.726 |
| *Cladonia stellaris* |  |  |  |  |  |  | 4 / 2 | 0.329 |  | 0.3 / 0.1 | 0.114 |  | **8 / 2** | **0.007** |  | 2.0 / 0.1 | 0.072 |  |  |  |  |  |  |
| *Cladonia ecmocyna/gracilis* |  |  |  |  |  |  | 5 / 2 | 0.160 |  | 0.3 / 0.1 | 0.215 |  | 9 / 9 | . |  | 0.7 / 0.3 | 0.124 |  | 10 / 7 | 0.127 |  | 0.2 / 0.1 | 0.128 |
| *Cladonia uncialis* |  |  |  |  |  |  |  |  |  |  |  |  | 6 / 6 | . |  | 1.4 / 0.3 | 0.226 |  | 7 / 5 | 0.392 |  | 0.2 / 0.3 | 0.645 |
| *Cladonia coc/ple* |  |  |  |  |  |  |  |  |  |  |  |  | **8 / 2** | **0.007** |  | 0.4 / 0.1 | 0.076 |  |  |  |  |  |  |
| *Cladonia chlorophaea* |  |  |  |  |  |  |  |  |  |  |  |  | **6 / 1** | **0.019** |  | **0.2 / 0.1** | **0.006** |  | 5 / 1 | 0.056 |  | 0.1 / 0.0 | 0.087 |
| *Cladonia deformis* |  |  |  |  |  |  |  |  |  |  |  |  | **8 / 0** | **<0.001** |  | **0.3 / 0.0** | **0.012** |  | 4 / 3 | 0.647 |  | 0.1 / 0.1 | 0.578 |
| *Nephroma arcticum* |  |  |  |  |  |  |  |  |  |  |  |  |  |  |  |  |  |  | 7 / 7 | . |  | 1.3 / 1.1 | 0.783 |
| *Cladonia carneola* |  |  |  |  |  |  |  |  |  |  |  |  |  |  |  |  |  |  | 5 / 2 | 0.170 |  | 0.1 / 0.1 | 0.187 |
| *Peltigera apthosa* |  |  |  |  |  |  |  |  |  |  |  |  |  |  |  |  |  |  | 5 / 5 | . |  | 0.5 / 0.7 | 0.483 |
| *Stereocaulon paschale* |  |  |  |  |  |  |  |  |  |  |  |  |  |  |  |  |  |  | 5 / 3 | 0.375 |  | 5.9 / 0.1 | 0.086 |
| *Peltigera scabrosa* |  |  |  |  |  |  |  |  |  |  |  |  |  |  |  |  |  |  | **4 / 0** | **0.027** |  | 0.3 / 0.0 | 0.101 |
| *Cladonia sulphurina* |  |  |  |  |  |  |  |  |  |  |  |  |  |  |  |  |  |  | **0 / 4** | **0.027** |  | 0.0 / 0.2 | 0.110 |

**Appendix S9.** Shifts in weighted means of relative proportions of C-, S- and R-strategies of plant communities in each plot between original survey and resurvey on a) fertile sites and b) infertile sites. C-, S- and R-strategy values were assigned for each species after Pierce et al. (2017), and community weighted means of each strategy class were calculated using species abundances in each plot.

**
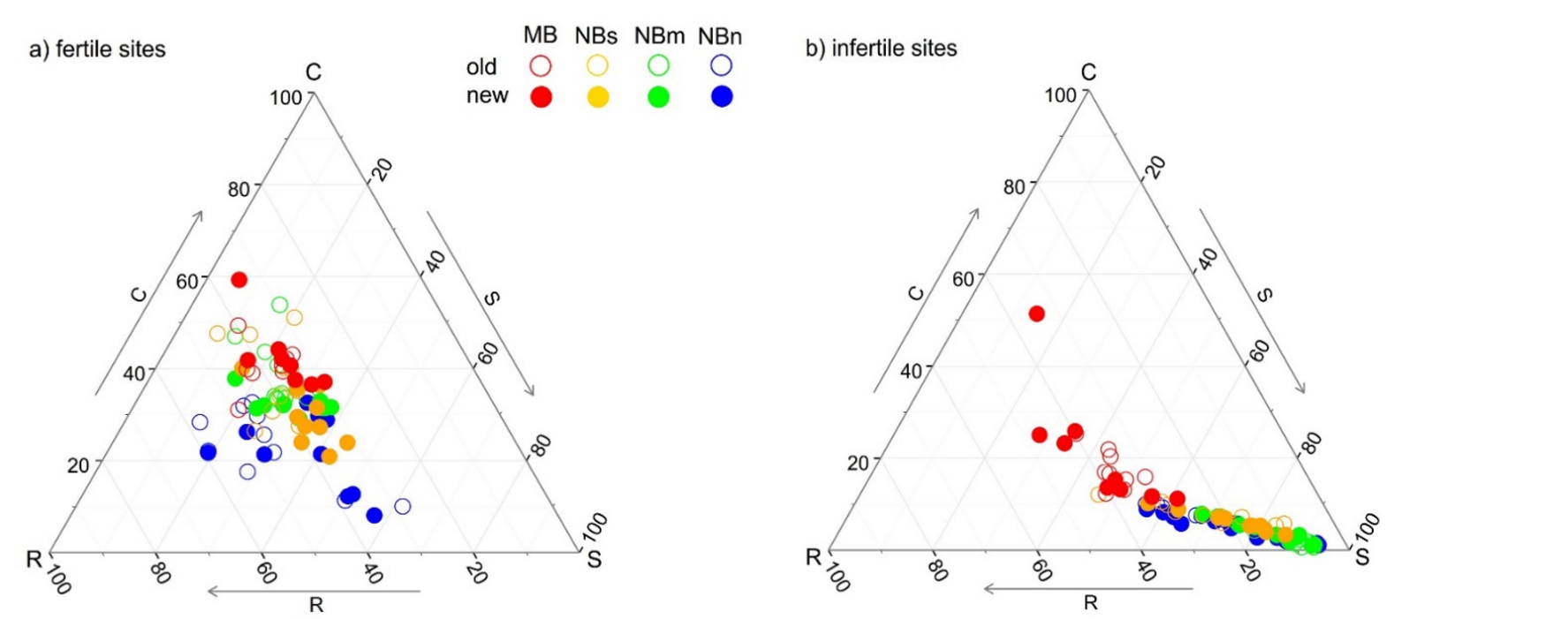
**

**Appendix S10. a)** Simulated lichen diversity for infertile sites and **b)** total species diversity for fertile and infertile sites with 95 % credible intervals. Diversity measures are effective numbers of Simpson diversity. The observed means of each subregion during the original survey and resurvey are indicated as gray-scale lines. **c)** Plot mean ± SE for effective number of species calculated for Simpson diversity measures (q = 2) in original survey (old) and resurvey (new) in fertile and infertile sites for each subregion. Raw species richness (q = 0) is indicated below each diversity measure. Note that raw species richness is not directly comparable between sites due to variation in sample plot size.

**a)
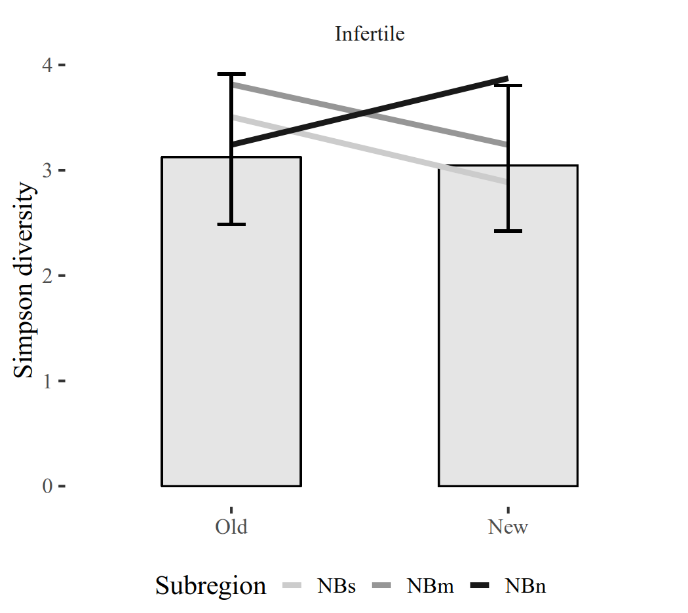
 b)
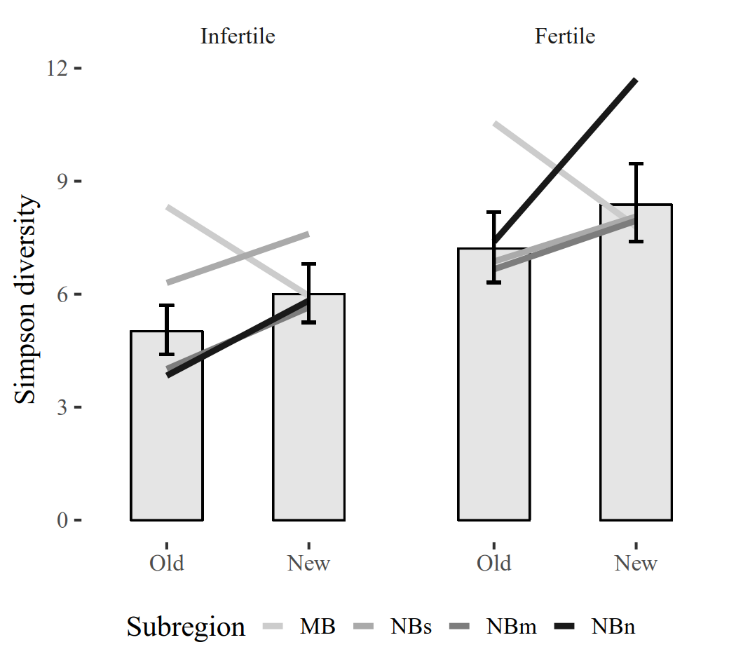
**

|  |  | total species diversity | |  | vascular plant diversity | |  | bryophyte diversity | |  | lichen diversity | |
| --- | --- | --- | --- | --- | --- | --- | --- | --- | --- | --- | --- | --- |
| **FERTILES** |  | old | new |  | old | new |  | old | new |  | old | new |
| MB | q = 2 | 10.6 ± 1.5 | 7.8 ± 1.3 |  | 8.3 ± 1.2 | 7.5 ± 1.2 |  | 2.8 ± 0.6 | 2.6 ± 0.5 |  |  |  |
|  | q = 0 | 30.8 ± 2.9 | 32.3 ± 3.7 |  | 26.3 ± 2.5 | 28.0 ± 3.0 |  | 4.5 ± 0.8 | 4.3 ± 1.0 |  |  |  |
| NBs | q = 2 | 6.9 ± 0.9 | 8.1 ± 0.8 |  | 4.9 ± 0.6 | 8.8 ± 0.9 |  | 2.8 ± 0.4 | 2.1 ± 0.2 |  |  |  |
|  | q = 0 | 33.8 ± 1.8 | 39.5 ± 2.0 |  | 28.2 ± 1.7 | 29.3 ± 1.0 |  | 5.6 ± 0.7 | 10.0 ± 1.3 |  |  |  |
| NBm | q = 2 | 6.7 ± 0.6 | 8.0 ± 0.8 |  | 5.1 ± 0.4 | 5.8 ± 0.6 |  | 1.9 ± 0.3 | 4.4 ± 0.6 |  |  |  |
|  | q = 0 | 26.8 ± 1.4 | 25.2 ± 0.8 |  | 20.5 ± 0.8 | 18.0 ± 1.1 |  | 6.3 ± 0.7 | 7.2 ± 0.9 |  |  |  |
| NBn | q = 2 | 7.4 ± 0.7 | 11.7 ± 0.9 |  | 6.2 ± 0.7 | 10.0 ± 1.0 |  | 1.8 ± 0.2 | 3.0 ± 0.5 |  |  |  |
|  | q = 0 | 34.1 ± 2.1 | 49.2 ± 2.1 |  | 28.4 ± 1.9 | 39.3 ± 2.1 |  | 4.8 ± 0.6 | 9.3 ± 0.8 |  |  |  |
| **INFERTILES** | |  |  |  |  |  |  |  |  |  |  |  |
| MB | q = 2 | 8.3 ± 1.4 | 6.0 ± 0.5 |  | 5.8 ± 1.2 | 3.8 ± 0.7 |  | 3.5 ± 0.4 | 3.5 ± 0.4 |  | 0.7 ± 0.3 | 0.0 ± 0.0 |
|  | q = 0 | 32.3 ± 3.9 | 27.6 ± 2.7 |  | 20.4 ± 3.0 | 17.0 ± 1.6 |  | 11.2 ± 1.0 | 10.4 ± 1.2 |  | 0.7 ± 0.3 | 0.0 ± 0.0 |
| NBs | q = 2 | 6.7 ± 0.8 | 7.7 ± 0.5 |  | 4.7 ± 0.5 | 3.6 ± 0.3 |  | 2.8 ± 0.2 | 4.6 ± 0.5 |  | 2.8 ± 0.8 | 2.0 ± 0.8 |
|  | q = 0 | 23.9 ± 1.3 | 28.2 ± 1.7 |  | 13.0 ± 0.9 | 13.2 ± 1.1 |  | 7.1 ± 0.5 | 12.4 ± 1.0 |  | 3.8 ± 1.2 | 2.6 ± 1.1 |
| NBm | q = 2 | 5.2 ± 0.4 | 6.0 ± 0.4 |  | 3.2 ± 0.4 | 3.5 ± 0.2 |  | 2.2 ± 0.3 | 2.8 ± 0.2 |  | 3.8 ± 0.3 | 2.9 ± 0.4 |
|  | q = 0 | 25.6 ± 1.0 | 22.3 ± 1.0 |  | 9.3 ± 1.6 | 8.5 ± 1.1 |  | 7.0 ± 0.6 | 8.6 ± 0.5 |  | 9.3 ± 0.8 | 5.2 ± 0.8 |
| NBn | q = 2 | 4.5 ± 0.3 | 6.1 ± 0.8 |  | 2.1 ± 0.1 | 4.0 ± 0.6 |  | 2.2 ± 0.3 | 2.5 ± 0.3 |  | 3.2 ± 0.7 | 3.8 ± 0.6 |
|  | q = 0 | 23.4 ± 1.3 | 28.4 ± 1.9 |  | 9.8 ± 0.9 | 12.9 ± 1.7 |  | 4.7 ± 0.4 | 8.5 ± 0.5 |  | 8.8 ± 1.2 | 7.0 ± 1.5 |

**c)**
